# Multi-omics-informed hydrogel design: modulating IL-6 to reduce endoplasmic reticulum stress in bone regeneration

**DOI:** 10.1016/j.bioactmat.2025.09.005

**Published:** 2025-10-15

**Authors:** Jiannan Zhou, Jingtao Dai, Shixian Hu, Cancan Qi, Jiahao Chen, Wentai Zhang, Dorothea Alexander, An Li, Yin Xiao, Ping Li

**Affiliations:** a School and Hospital of Stomatology, Guangdong Engineering Research Center of Oral Restoration and Reconstruction & Guangzhou Key Laboratory of Basic and Applied Research of Oral Regenerative Medicine, Guangzhou Medical University, Guangzhou, China; b Department of Orthodontics, Stomatological Hospital, School of Stomatology, Southern Medical University, Guangzhou, China; c Institute of Precision Medicine, the First Affiliated Hospital, Sun Yat-Sen University, Guangzhou, China; d Microbiome Medicine Center, Department of Laboratory Medicine, ZhuJiang Hospital, Southern Medical University, Guangzhou, China; e Department of Prosthodontics, Geriatric Dentistry and Craniomandibular Disorders, Charité-Universitätsmedizin Berlin, Corporate Member of Freie Universität Berlin and Humboldt-Universität zu Berlin, Berlin, Germany; f Dongguan Key Laboratory of Smart Biomaterials and Regenerative Medicine, The Tenth Affiliated Hospital of Southern Medical University, Dongguan, Guangdong, China; g Department of Oral and Maxillofacial Surgery, University Hospital Tübingen, Tübingen, Germany; h Department of Periodontology, Stomatological Hospital, School of Stomatology, Southern Medical University, Guangzhou, China; i School of Medicine and Dentistry, Institute for Biomedicine and Glycomics, Griffith University, Gold Coast, Queensland, Australia

**Keywords:** Bone regeneration, Multi-omics, Endoplasmic reticulum stress, Interleukin-6, Hydrogel

## Abstract

Post-traumatic bone healing exhibits significant heterogeneity, especially in different injury sites. Notably, bone healing progresses more rapidly in alveolar bone defects compared to the slower repair process observed in femoral bone. Given this physiological phenomenon, understanding site-specific differences is crucial for designing functional biomaterials to enhance bone regeneration. This study, via multi-omics analysis, identified the pivotal role of high interleukin-6 (IL-6) expressing alternatively activated (M2) macrophages in early alveolar bone healing. It was found that IL-6 level in M2 macrophages could modulate heat shock protein family A member 5, alleviating endoplasmic reticulum stress (ERS) and preventing apoptosis, thereby promoting bone regeneration. Based on these findings, a gelatin-based porous hydrogel optimized for localized IL-6 delivery was further developed to accelerate bone healing in femoral defects. The results demonstrated that this hydrogel significantly enhanced femoral bone regeneration by modulating ERS and hematoma responses. These findings offer promising strategies for enhancing bone regeneration.

## Highlights

• Multi-omics strategy advanced tailored material design.
• Multi-omics analysis revealed IL-6's role in alveolar bone healing.
• IL-6 in M2 macrophages alleviated ERS and prevented apoptosis.
• Gelatin-based hydrogel was optimized for localized IL-6 delivery.
• Hydrogel enhanced femoral bone regeneration by modulating ERS.

## Graphical abstract

**Figure:**
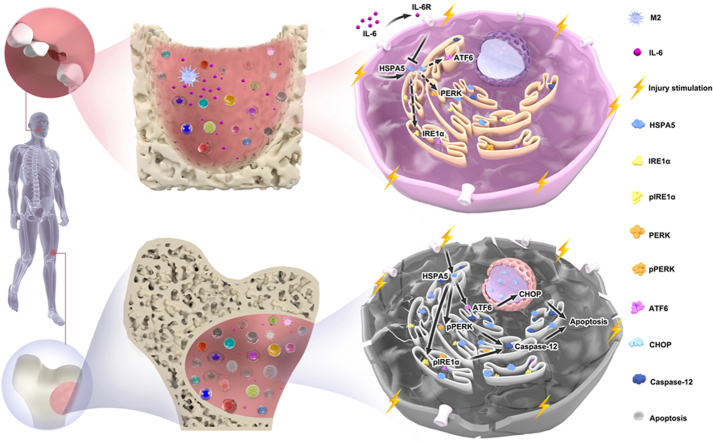

## 1 Introduction

Bone defects and injuries occur across all age groups and pose a significant global health challenge [1,2]. While bones inherently possess the capacity for self-repair, the process is influenced by defect size, location, age, systematic disorders, and infections, which all result in different bone healing microenvironments. Notably, bone healing varies significantly across anatomical sites, suggesting site-specific mechanisms [3]. For instance, rapid healing is observed in tooth extraction sockets and alveolar bone defects [4]. In contrast, femoral bone tissue exhibits slower healing, indicating distinct site regulatory mechanisms govern the complex bone repair processes [5]. Inspired by this physiological phenomenon, it is crucial to understand site-specific variations to guide the design of biomaterials that enhance bone repair.

Post-traumatic bone healing is a complex, precisely coordinated process. The process begins with the formation of a blood clot and hematoma, followed by an inflammatory phase [6,7]. This decisive early phase is critical since disruptions during this period can delay or compromise bone regeneration success [8]. The healing process triggers an inflammatory stress reaction that depends on meticulous spatial and temporal regulation [9,10]. The injured callus contains a dynamic cellular composition that continuously adapts to environmental changes [11]. Various immune cells, including macrophages, T lymphocytes, B lymphocytes, mast cells, and neutrophils, are crucial to bone healing. The subsequent healing phases are characterized by sequential, interconnected, and overlapping processes, each comprising distinct cellular compositions essential for the progression of healing. Dysregulation of this inflammatory stress cascade detrimentally affects subsequent healing phases, impeding overall progress [12,13].

Multi-omics technologies merge data from genomics, transcriptomics, proteomics, and metabolomics, offering a promising approach to understanding cellular responses and their interactions within tissue microenvironments [14,15]. This comprehensive analysis enhances our understanding; however, employing single-cell omics to investigate bone tissue poses challenges. The primary difficulty lies in the preparation of high-quality single-cell suspensions from bone tissue. Also, the multi-omics technologies have been employed to analyze cellular mechanisms, with the insights gained subsequently informing the design of relevant biomaterials. [16]. For example, Lin et al. reported utilizing single-cell RNA sequencing (scRNA-seq) to develop and optimize a multi-enzymatic hydrogel scaffold to promote self-regenerative repair in diabetic mandibular defects by reprogramming macrophages and rejuvenating bone marrow-derived mesenchymal stem cells and endothelial cells [16]. Nevertheless, there remains a gap in dynamic, comprehensive studies that detail cellular activities during the early stages of bone repair across various sites to guide the design of functional bone biomaterials.

**Scheme 1:**
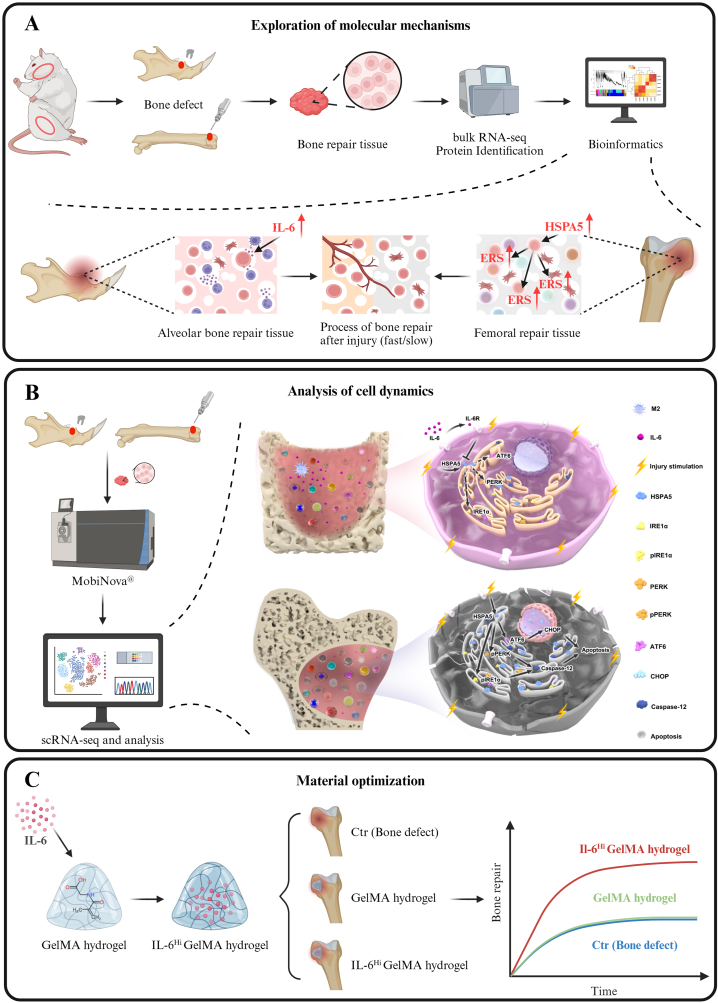
Schematic illustration of multi-omics approaches to guide materials design: (A) Exploring molecular mechanisms behind physiological phenomena. (B) Analyzing single-cell dynamics for crucial biological cues. (C) Optimizing material design to improve bioactivity.

In this study, multi-omics technologies were used to examine the dynamic behavior of immune cells during the critical early phase of bone healing following injuries to alveolar and femoral bones. By integrating transcriptomics and protein identification data, critical biological insights were extracted that guided the design of innovative materials (Scheme 1). Specifically, the dynamic behavior of immune cells during the critical early phase of bone healing after injuries to alveolar and femoral bones was characterized using transcriptomics and protein identification. Increased IL-6 secretion after alveolar injury was identified, which modulates Heat Shock Protein Family A (Hsp70) Member 5 (HSPA5), suppressing endoplasmic reticulum stress (ERS)-related apoptosis. Local desirable IL-6 delivery via a hydrogel could effectively promote bone regeneration and mitigate ERS-mediated apoptosis. These findings highlight the critical regulatory role of IL-6-generating macrophages in early-stage bone healing and propose a novel strategy for material design in bone repair.

## 2 Materials and methods

### 2.1 Animal models

Male Sprague-Dawley (SD) rats weighing 300 ± 5 g were used in this study (Beijing Vital River Laboratory Animal Technology Co., Ltd.). The study strictly adhered to the Animal Research: Reporting of *in vivo* Experiments guidelines. The animal protocol employed in this investigation was approved by the local animal ethics committee (Number: GY2023-721). All rats had unrestricted access to food and water. They were housed in a specific pathogen-free barrier environment. The ambient temperature was maintained at 22–24 °C, with a relative humidity of 50–70 % and a 12-h light/12-h dark cycle. To establish a common alveolar bone defect model, the maxillary first molars of the rats were extracted. Additionally, surgical procedures were carried out to create femoral defects in rats, as previously reported [17]. Specifically, defects with a diameter and depth of 3.5 mm were made at the medial epicondyle of the femur in nine rats. Following the femoral defect creation, HA15 (HY-100437, MCE, US), LMT28 (HY-102084, MCE, US), HM03 (HY-125974, MCE, US), and SC144 (HY-15614, MCE, US) were administered to the rats via intraperitoneal injection, in accordance with the manufacturer's instructions. A group size of at least 18 animals was used per experimental condition to ensure robust biological replication. All animal samples were selected in compliance with the 3R principles (Replacement, Reduction, Refinement) and guided by statistical power requirements.

### 2.2 Histological staining

Freshly isolated femoral and alveolar bone tissues were rinsed with pre-cooled phosphate-buffered saline (PBS) and fixed overnight at 4 °C in 4 % paraformaldehyde. Following fixation, the samples underwent decalcification using 10 % ethylenediaminetetraacetic acid (EDTA, pH 7.4) under gentle agitation at room temperature until the bone matrix achieved sufficient softening for subsequent histological analysis. Subsequently, the samples were dehydrated and embedded in paraffin. Tissue sections (3 μm in thickness) were cut using a paraffin microtome (HistoCore BIOCUT, Leica) and mounted on adhesive glass slides for further processing.

For hematoxylin and eosin (H&E) staining, the tissue sections were dehydrated and dewaxed in graded ethanol solutions. The tissue sections were then stained with hematoxylin and eosin according to the manufacturer's instructions, dehydrated, and mounted with neutral resin. Pathological changes in the repair tissues were observed under an optical microscope (DM2500, Leica).

Regarding staining procedures using the Masson staining kit (BP-DL641, Sbjbio, CN), Von Kossa staining kit (WLA154, Wanleibio, CN), reactive oxygen species (ROS) assay kit (BB-470516, Bestbio, CN), and methylene blue acid fuchsin staining solution kit (C0189S, Beyotime, CN), staining was performed according to the respective manufacturers' protocols. Observations were made under an optical microscope (DM2500, Leica).

Regarding immunohistochemistry (IHC) staining, antigen retrieval was conducted using a citrate buffer solution after dehydration and dewaxing. IHC staining involved inhibiting endogenous peroxidase activity with hydrogen peroxide solution and blocking the sections with a 3 % bovine serum albumin solution for 30 min. Primary antibodies (Bioss: HSPA5, bs-1219R; IRE1, bs-8680R; phospho-IRE1α, bs-4308R; PERK, bs-2469R; phospho-PERK, bs-3330R; ATF6, bs-1634R; Caspase-12, bs-0967R; FHOD3, bs-13156R, CN; Proteintech: CHOP, 15204-1-AP, CN; Thermo Fisher Scientific: IL-6, MA5-51298, US) were added and incubated overnight at 4 °C, followed by incubation with secondary antibodies for 1 h. Nuclei were stained with DAPI reagent (C1005, Beyotime, CN), and the sections were mounted with glycerol. The samples were observed and photographed using an upright fluorescence microscope (DM2500, Leica). The proportion of positive staining was analyzed using ImageJ (Version 1.8.0) statistical software.

### 2.3 Micro-computed tomography analysis

All the rat femurs and alveolar bones were fixed overnight in 4 % paraformaldehyde and washed with PBS before scanning. The samples were imaged using a micro-computed tomography (μCT) scanner (NEMO micro-CT, Pingseng Scientific) with 90 kVp tube voltage and 80 μA current. Three-dimensional (3D) segmentation of the μCT images was conducted using commercial image processing software (Recon, Pingseng Scientific). Each scanned image was evaluated using a consistent threshold value for the 3D structural rendering of each sample. As reported in previous studies, the 3D histomorphometric analysis encompassed several parameters: bone mineral density (BMD) of cartilage, the bone volume-to-tissue volume (BV/TV) ratio, trabecular spacing (Tb.Sp), and trabecular thickness (Tb.Th) [18]. For each specimen, an uninjured contralateral site (either the intact contralateral femur or alveolar bone) was chosen as an internal control. This approach accounted for the intersubject anatomical variability between alveolar and femoral bone. The postoperative micro-CT parameters (BMD, BV/TV, Tb.Th, Tb.Sp) at the defect site were normalized to the corresponding regions of the contralateral control. Subsequently, comparative analyses between alveolar bone and femoral defects were carried out using these standardized values.

### 2.4 Bulk RNA-seq and data analysis

Total RNA was extracted using TRIzol reagent under the manufacturer's instructions. RNA purity and quantity were assessed using a spectrophotometer (NanoDrop 2000, Thermo Scientific), while RNA integrity was evaluated with a bioanalyzer (Agilent 2100, Agilent Technologies, Santa Clara). Transcriptome libraries were constructed using the VAHTS Universal V6 RNA-seq Library Prep Kit according to the protocol provided, which were then sequenced on an Illumina Novaseq 6000 sequencing platform, generating 150 bp paired-end reads. Raw reads in fastq format were processed using fastp software to eliminate low-quality reads, yielding high-quality reads for subsequent data analyses [19]. HISAT2 software was employed for reference genome alignment, and gene expression levels were quantified [20]. Read counts for each gene were generated using HTSeq-count [21]. Principal component analysis (PCA) analysis and plotting of gene counts were performed using R (Version 3.2.0) to assess biological replicates across samples.

Differentially expressed genes (DEGs) were identified using DESeq2 software, employing a significance threshold of *p* < 0.05 and a fold change >1.5 [22]. Hierarchical clustering analysis of DEGs was conducted using R (Version 3.2.0) to visualize expression patterns in different groups and samples. Kyoto Encyclopedia of Genes and Genomes (KEGG) and Gene Ontology (GO) pathway enrichment analyses were performed on the DEGs using the hypergeometric distribution algorithm to screen for significantly enriched functional categories [23,24]. The “WGCNA” package was used to construct networks and identify relevant modules [25].

### 2.5 Real-time quantitative PCR

RNA was extracted using an RNA extraction kit (EZB-RN4, EZBioscience, US). Total RNA was isolated with the GoScript™ Reverse Transcription System Kit (A2800, Promega, US), and post-transcription cDNA synthesis was performed for real-time quantitative polymerase chain reaction (RT-qPCR) using SYBR Premix Ex Taq™II (AG11701, AG, CN) under the instructions and the previous experimental methods [26]. Supplementary Table presents the primer sequences.

### 2.6 Protein identification and data analysis

Early repair tissues of femoral and alveolar bone defects underwent a peptide identification procedure using the Orbitrap Exploris 480 mass spectrometer. Specifically, 64,890 spectra were acquired from femoral bone samples, 13,541 of which were successfully matched and identified through the search engine, with 1584 proteins and 9449 peptides identified. Concerning the alveolar bone, 66,203 spectra were generated, with 17,746 spectra successfully matched and identified, yielding 2352 proteins and 14,277 peptides. Subsequently, highly abundant proteins were extracted and subjected to GO and KEGG pathway enrichment analyses to filter out significantly enriched functional terms. Furthermore, the String database and Cytoscape software were utilized to visualize protein-protein interaction networks [27,28].

### 2.7 Western blotting

Total proteins were extracted using RIPA lysis buffer (P0013B, Beyotime, CN), followed by collecting lysates and centrifugation at 4 °C (13,400 rpm for 15 min). The proteins were then separated and transferred onto PVDF membranes placed on 10 % SDS-PAGE gels (P0690, Beyotime, CN). The membranes were blocked in 5 % skimmed milk for 1 h and incubated overnight at 4 °C with primary antibodies (Bioss: HSPA5, bs-1219R; IL-6, bs-4539R; IRE1, bs-8680R; phospho-IRE1α, bs-4308R; PERK, bs-2469R; phospho-PERK, bs-3330R; ATF6, bs-1634R; Caspase-12, bs-1105R; RUNX2, bs-1134R; Osterix, bs-25532R, CN; Proteintech: CHOP, 15204-1-AP, CN). After three rounds of washing with TBST buffer, the membranes were incubated with HRP-conjugated secondary antibodies for 1 h at room temperature. Subsequently, the bound antibodies were detected using the Odyssey infrared imaging system (Odyssey® DLx, LI-COR). The band intensities were analyzed using ImageJ software (Version 1.8.0).

### 2.8 scRNA-seq

Defect models were established in the femurs and alveolar bones of male SD rats, and repair tissues were collected from the bone defects after four days for scRNA-seq. Specifically, the GEM Single Cell 3′ kit was used for GEM generation and barcoding, followed by post-GEM-RT clean-up and cDNA amplification, and subsequent 3′ gene expression library construction before sequencing. The MobiVision software pipeline (Version 1.1), provided by MobiDrop, was used to demultiplex cellular barcodes, align reads to the genome and transcriptome using the STAR aligner, and downsample reads as necessary to generate normalized aggregate data across samples. This process produced a matrix comprising gene counts versus cells, facilitating a comprehensive analysis of the transcriptional landscape within the repair tissues.

### 2.9 scRNA-seq data processing and analysis

The R package “Seurat” was used for quality control, dimensionality reduction, and clustering of scRNA-seq data [29]. Cells with a gene count <6000 and >200 and a mitochondrial gene fraction of <25 % were deemed high-quality and included in subsequent analyses. Following normalization, the FindVariableFeatures() function is used to select highly variable genes (*p* < 0.05). Principal component analysis and uniform manifold approximation and projection (UMAP) were then performed for dimensionality reduction and visualization. Subsequently, cell populations were clustered and identified based on canonical cell markers. The FindAllMarkers() function in the Seurat package was used for differential gene expression analysis. The CellChat package was used to analyze cell-cell communication within distinct subpopulations [30]. The Slingshot package was used for pseudotime analysis [31]. Lastly, the SangerBox website was used to visualize KEGG and GO enrichment analyses of DEGs [32].

### 2.10 Synthesis and characterization of GelMA hydrogel

Lyophilized gelatin methacryloyl (GelMA, 0.1 g) was dissolved in deionized water with L-alanine phosphate (1.0 mL, 0.2 % w/v) at 50 °C in the dark, then sterilized via 0.22 μm filtration. IL-6 was incorporated into the sterile GelMA solution and stirred for 1 h in the dark prior to 405 nm photo-crosslinking. Hydrogel morphology was analyzed by SEM. Rheological moduli (G′, G″) were measured at 37 °C using a TA rheometer (1.0 Hz, 1 rad/s). For the swelling analysis, the UV-polymerized discs were dried and weighed (W_d_), immersed in PBS (pH = 7.4, 37 °C), removed at designated time points, blotted dry with filter paper to remove surface moisture, and then weighed to obtain the wet weight (W_w_). The swelling ratio was calculated using the formula: (W_w_ -W_d_)/W_d_ × 100 %. Hydrolytic degradation was monitored for 6–96 h using uniformly fabricated hydrogel discs.

### 2.11 Enzyme-linked immunosorbent assay

The levels of IL-6 in the early stages of femoral and alveolar bone defects and the release levels of IL-6-loaded GelMA hydrogels (GelMA^IL−6^) at various time intervals were assessed under the instructions provided in the ELISA detection kit (EK306, Multi Sciences, CN). The tissues designated for testing were weighed, homogenized, diluted in proportion, frozen, and thawed three times at −80 °C. After centrifugation at 800 rpm for 5 min, 100 μL of the supernatant was added to the detection well plate. After a 2-h incubation at 37 °C, the absorbance was measured using an enzyme marker (Varioskan ALF, Thermo Scientific), and IL-6 levels were calculated.

### 2.12 Calcein-AM/PI staining

According to the instructions and the previous experimental methods, fluorescence analyses were performed on both GelMA^IL−6^ hydrogel-stimulated and -unstimulated HUVECs (CBP60340, Cobioer, CN) and HCFs (SNP-H320, Sunncell, CN) using the Calcein-AM/PI Double Stain Kit (CA1630, Solarbio, CN) [33]. Living cells were visualized by calcein-AM, emitting green fluorescence, while PI was used to stain dead cells, which were subsequently visualized in the red channel.

### 2.13 Multiple immunofluorescence

For immunofluorescence (IF) co-staining, a multiplex fluorescence immunohistochemistry kit (pH 9.0, RS0037, ImmunoWay, US) was utilized strictly following the manufacturer's instructions. The antibodies were applied in a sequential manner. First, HSPA5 (bs-1219R, Bioss, CN) was used, followed by IL-6 (MA5-51298, Thermo Fisher Scientific, US), ARG1 (bsm-56207R, Bioss, CN), FHOD3 (bs-13156R, Bioss, CN), RUNX2 (bs-1134R, Bioss, CN), and Osterix (YA3539, MCE, US). After antibody staining, nuclear counterstaining was performed using DAPI. To analyze the co-localization of staining markers, ImageJ software was employed.

### 2.14 Detection of DNA fragmentation

The terminal deoxynucleotidyl transferase dUTP nick-end labeling (TUNEL) assay was employed to detect DNA fragmentation associated with cell death. For TUNEL-based apoptosis detection in decalcified, paraffin-embedded femoral and alveolar bone tissues, the following steps were carried out. First, the tissue sections were deparaffinized in xylene, rehydrated through a graded ethanol series (100 %, 95 %, 80 %, 70 %), and then rinsed in distilled water. Next, permeabilization was achieved by treating the sections with 20 μg/mL proteinase K at 37 °C for 15–20 min, followed by two washes with PBS. The TUNEL reaction mixture, which was prepared according to the manufacturer's instructions for the TUNEL apoptosis detection kit (E-CK-A320, Elabscience, CN) and contained terminal deoxynucleotidyl transferase (TdT) enzyme and fluorescein-dUTP, was applied to the sections. The sections were then incubated in a humidified chamber at 37 °C for 60 min, protected from light. After three rinses with PBS, the nuclei were counterstained with DAPI for 5 min. Finally, the fluorescence signals were visualized using an upright fluorescence microscope.

### 2.15 Statistical analyses

All data are presented as mean ± standard deviation, based on a minimum of three independent experiments for each study. Statistical analyses were performed using either a two-tailed Student's t-test or a one-way analysis of variance (ANOVA) with post hoc comparisons. Graphs were generated using GraphPad 9 software (Version 9.0). Each experiment was performed in triplicate. Differences were considered statistically significant at *p* < 0.05.

## 3 Results

### 3.1 Rapid early repair in alveolar bone compared to femoral bone

**Fig. 1:**
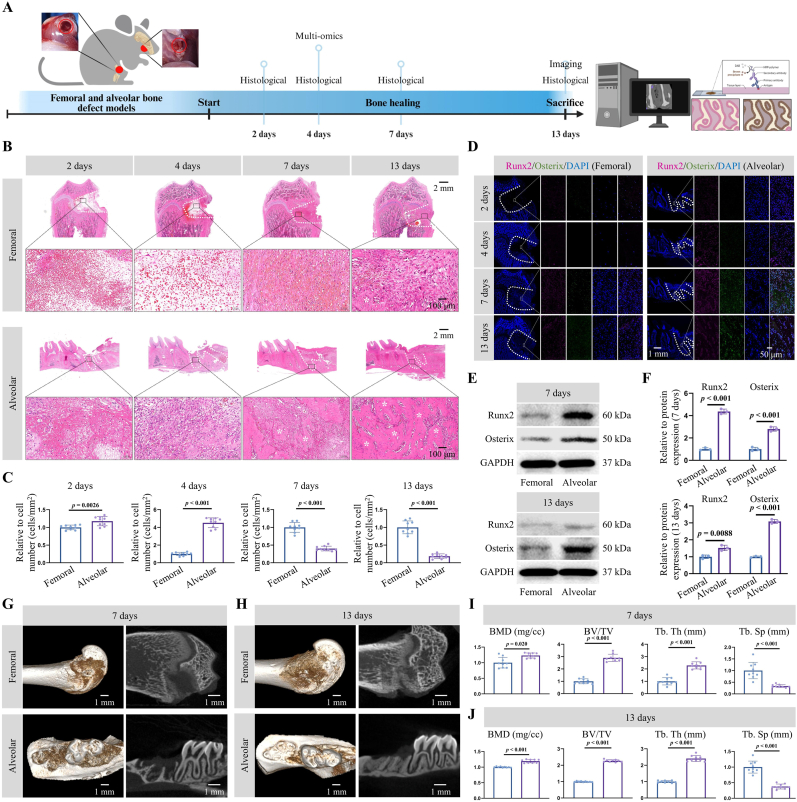
Comparative analysis of the early repair processes of femoral and alveolar defects. (**A**) Overview of the research timeline after preparing rat bone defect models. (**B**, **C**) Hematoxylin and eosin staining and its quantitative analysis of early repair tissue between femoral and alveolar defects at different time intervals. Scale bars: 2 mm and 100 μm *, bone tissue. (**D**) Immunofluorescence staining of Runt-related transcription factor (Runx2) and Osterix proteins in early-stage repair tissues of femoral and alveolar bone defects at different time intervals. Scale bars: 1 mm and 50 μm. (**E**, **F**) Western blot detection and statistical analysis of Runx2 and Osterix protein expression in repair tissues from femoral and alveolar bone at 7 days and 13 days. (**G**–**J**) Micro-CT analysis of femoral and alveolar defects on days 7 and 13. Scale bar: 1 mm. Parameters include bone mineral density (BMD), bone volume fraction (BV/TV), trabecular thickness (Tb.Th), and trabecular separation (Tb.Sp). Statistical significance was assessed using a *t*-test.

To investigate the changes at various time intervals following femoral and alveolar bone defects, maxillary first molars were extracted from rats to establish an alveolar bone defect model while similar size bone defects were concurrently created in the same animal at the medial epicondyle of the femur (Fig. 1A). Regarding histological characteristics, H&E staining showed that the repair tissue in the femur was predominantly composed of blood-related cells 2 days after defect creation, whereas the alveolar bone exhibited fewer blood cells and the presence of collagen fiber-like tissue (Fig. 1B). On day 4, the femur exhibited a reduced number of blood cells and the appearance of collagen fiber-like tissue. Simultaneously, the alveolar bone's collagen fiber-like tissue became denser and was associated with partial lymphocyte infiltration (Fig. 1B). On day 7, dense collagen fiber-like tissue was observed in the femur, while small patches of osteoid tissue emerged in the alveolar bone (Fig. 1B). On day 13, small patches of osteoid tissue began to emerge in the femur, while the alveolar bone displayed dense ossification (Fig. 1B). An analysis of cell counts from H&E indicated a significant increase in alveolar bone cell numbers on day 4, followed by a subsequent decrease. In contrast, femoral cell numbers gradually increased (Fig. 1C). Notable differences in regenerative capacity between femoral and alveolar bone were evident by day 4 post-defect creation.

To further investigate the aforementioned observations, repair tissues from the defect sites were harvested and subjected to Masson's trichrome staining and methylene blue-acid fuchsin staining (Fig. S1). The results aligned with the H&E staining analysis, revealing that, by day 13, the callus in the alveolar bone exhibited greater maturity than that in the femur (Fig. S1A–D). Furthermore, statistical analysis was performed on Masson's trichrome and methylene blue-acid fuchsin staining across different time intervals following detection of the femoral and alveolar bone defects (Fig. S1E). These results revealed that, starting from the fourth day after injury, obvious differences emerged between the femur and alveolar bone.

To characterize these differences, the localization and expression patterns of the osteogenic marker proteins Runx2 and Osterix in the femur and alveolar bone were examined separately (Fig. 1D and Fig. S1F). The results demonstrated that Runx2 protein expression began in the alveolar bone on day 4, whereas the femur showed only minimal expression. By day 7, Osterix expression was initiated in the alveolar bone. Its expression intensified significantly by day 13. In contrast, both proteins displayed minimal expression in the femur throughout this period. In line with these observations, Western Blot analysis demonstrated a marked elevation in the expression of Runx2 and Osterix proteins in the alveolar bone compared to the femur at 7 and 13 days post-injury (Fig. 1E and F).

The results of μCT revealed bone regeneration in both femoral and alveolar bones on day 7. The alveolar bone showed significantly greater the values of BMD, BV/TV, and Tb.Th. Conversely, the value of Tb.Sp in the alveolar bone was notably lower than that in the femur (Fig. 1G–I). The bone regeneration patterns in both femoral and alveolar bones closely resembled the trends observed on day 7. Notably, the alveolar bone exhibited a significantly more pronounced healing response (Fig. 1H–J).

### 3.2 Increased IL-6 expression during initial stages of alveolar bone repair

**Fig. 2:**
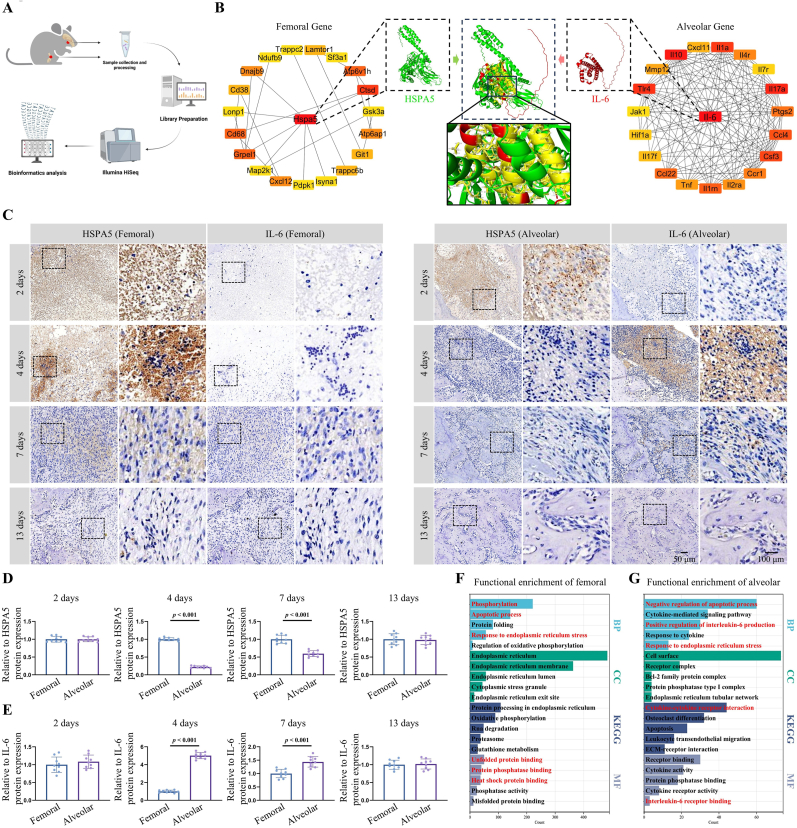
Bulk RNA-seq identification of early regulatory genes and signals in bone defects. (**A**) Experimental workflow for bulk RNA-seq in early stages of bone defects in rats. (**B**) Construction of interaction networks and molecular dynamics simulations for key regulatory proteins in early-stage femoral and alveolar bone defects. (**C**–**E**) Immunohistochemical detection and quantitative analysis of Heat Shock Protein Family A (Hsp70) Member 5 (HSPA5) and interleukin-6 (IL-6) expression levels in early healing tissues of the femoral and alveolar regions. Scale bars: 2 mm, 100 μm, and 50 μm. (**F**, **G**) Kyoto Encyclopedia of Genes and Genomes (KEGG) and Gene Ontology (GO) enrichment analysis results for differentially expressed genes (DEGs) in early healing stages of femoral and alveolar bone defects. Statistical significance was determined using a *t*-test.

Bulk RNA-seq was conducted on the repair tissues on day 4 to investigate the differences between the femur and alveolar bone in the early healing processes (Fig. 2A). The sequencing data revealed robust sample clustering and a distinct PCA distribution (Fig. S2A). Differential expression analysis was used to identify DEGs between femoral and alveolar bone defects, yielding 5933 and 1155 DEGs in the femur and alveolar bones, respectively (Fig. S2B). Sample-specific module enrichment was carried out for both bone types using weighted correlation network analysis (WGCNA), which revealed a significant positive correlation between “Red” module genes and the femur and between “Magenta” module genes and the alveolar bone (Fig. S2C). The DEGs were intersected with the WGCNA modules specific to each bone type to identify crucial genes involved in the early healing stages. The approach identified 152 and 190 shared genes for the femur and alveolar bone, respectively, during the early healing phase (Fig. S2D).

Using the “cytohubba” plugin, hub genes that may play pivotal roles in the early healing stages of femoral and alveolar bone defects were identified, with the results indicating that *Hspa5* in the femur and *Il-6* in the alveolar bone emerged as potential key nodes in the early healing process (Fig. 2B). RT-qPCR analysis revealed significant upregulation of *Hspa5* in the early stages of femoral defects compared to alveolar bone defects. However, *Il-6* showed a marked increase in the early stages of alveolar bone defects (Fig. S2E). While other screened genes, including *Cstd*, *Atp6v1h*, *Il-10*, and *Il-17α*, also exhibited some differences, the magnitude of these variations was inferior to that in the hub genes *Hspa5* and *Il-6* (Fig. S2E).

Immunohistochemistry was employed for histological characterization to assess the expression levels of the core genes *Hspa5* and *Il-6* at different time points during the early stage of the defect. At 2 days post-injury, HSPA5 protein expression was evident in both alveolar bone and femur, whereas IL-6 protein expression was relatively minimal (Fig. 2C–E). On day 4, distinct expression patterns of HSPA5 and IL-6 were observed between the femur and the alveolar bone. In the femur, HSPA5 maintained a high level of expression, whereas IL-6 showed only minimal expression. Conversely, in the alveolar bone, HSPA5 expression was significantly reduced, and IL-6 expression was markedly increased (Fig. 2C–E). Subsequently, on day 7, a decline in HSPA5 expression was observed in the femur, and IL-6 expression in the alveolar bone also showed a similar reduction. By day 13, the expression levels of both HSPA5 and IL-6 were notably low in both the alveolar bone and the femur (Fig. 2C–E). Also, the granulation tissues extracted from femoral and alveolar bone defects on day 4 post-injury demonstrated HSPA5 and IL-6 expression patterns consistent with the aforementioned findings (Fig. S2F). These findings indicated that HSPA5 and IL-6 critically regulated the early repair processes in both femoral and alveolar bone defects, predominantly exerting their effects around day 4 post-injury.

The KEGG and GO enrichment analyses were conducted on the genes above to further explore the early repair mechanisms of femoral and alveolar bone defects. The enrichment results in the early stages of femoral defects primarily included phosphorylation, apoptotic processes, ERS response, protein phosphatase binding, unfolded protein binding, and heat shock protein binding (Fig. 2F). Conversely, the early stages of alveolar bone defects exhibited significant enrichment of the negative regulation of apoptotic processes and ERS response, positive regulation of IL-6, cytokine response, and interleukin-6 receptor binding (Fig. 2G).

**Fig. 3:**
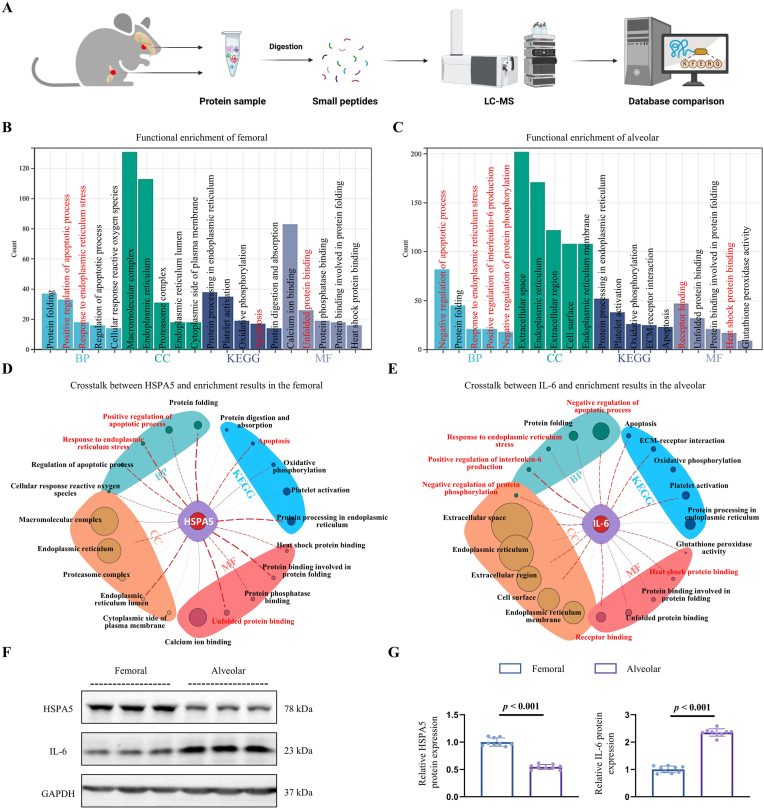
Protein identification for key proteins and signals in early bone defects. (**A**) Workflow for tissue protein identification at early stages of bone defects in rats. (**B**, **C**) KEGG and GO enrichment analysis of differential proteins in early healing tissues of femoral and alveolar bone defects. (**D**, **E**) Interaction network analysis of HSPA5 and IL-6 within the enrichment signals of femoral and alveolar bones. (**F**) Protein expression levels of HSPA5 and IL-6 in early healing tissues of femoral and alveolar defects. (**G**) Quantitative analysis of HSPA5 and IL-6 protein expression. Statistical analysis employed a *t*-test.

Using the aforementioned bulk RNA-seq data, protein identification methods were employed to investigate the proteomic landscape during the early stages of healing in femoral and alveolar bone defects to corroborate our initial findings (Fig. 3A). Various metrics, including unique spectrum distribution, unique peptide distribution, protein mass distribution, protein coverage distribution, and peptide length distribution were used for the quality assessment of the protein identification procedures (Fig. S3A–E). Sample-specific extraction was performed to dissect the protein profiles, revealing 427 and 721 unique proteins in early femoral and alveolar bone defect samples, respectively (Fig. S3F). Subsequently, these specific proteins from femoral and alveolar bones were analyzed using KEGG and GO enrichment analyses. The enrichment results revealed the bulk RNA-seq data, indicating that early femoral defects were predominantly associated with positive regulation of apoptotic processes, ERS response, oxidative phosphorylation, protein folding, and heat shock protein binding (Fig. 3B). In contrast, early alveolar bone defects were characterized by negative regulation of apoptotic processes, positive regulation of IL-6, and negative regulation of protein phosphorylation, ERS response, and binding of heat shock proteins (Fig. 3C).

The relationship between the critical gene-encoded proteins HSPA5 and IL-6 in the femur and alveolar bone and the enrichment outcomes was further investigated by constructing a protein-signaling pathway interaction network, in which the thickness of dashed lines indicated the strength of interactions. According to our findings, the HSPA5 protein, a key expression product during the early stages of femur defect, exhibited a robust association with ERS and its related responses (Fig. 3D). Conversely, the IL-6 protein, crucial in the early alveolar bone defect, was primarily implicated in the reverse regulation of ERS-related reactions (Fig. 3E). Additionally, proteins were isolated from the early reparative tissues of femur and alveolar bone defects to determine the expression levels of HSPA5 and IL-6. The protein expression profiles reinforced the IHC results mentioned above, demonstrating a significant upregulation of HSPA5 in the early femur defect and a similar but distinct elevation of IL-6 in the early alveolar bone defect (Fig. 3F and G).

### 3.3 Cellular profiling of bone defects in the early healing phase

**Fig. 4:**
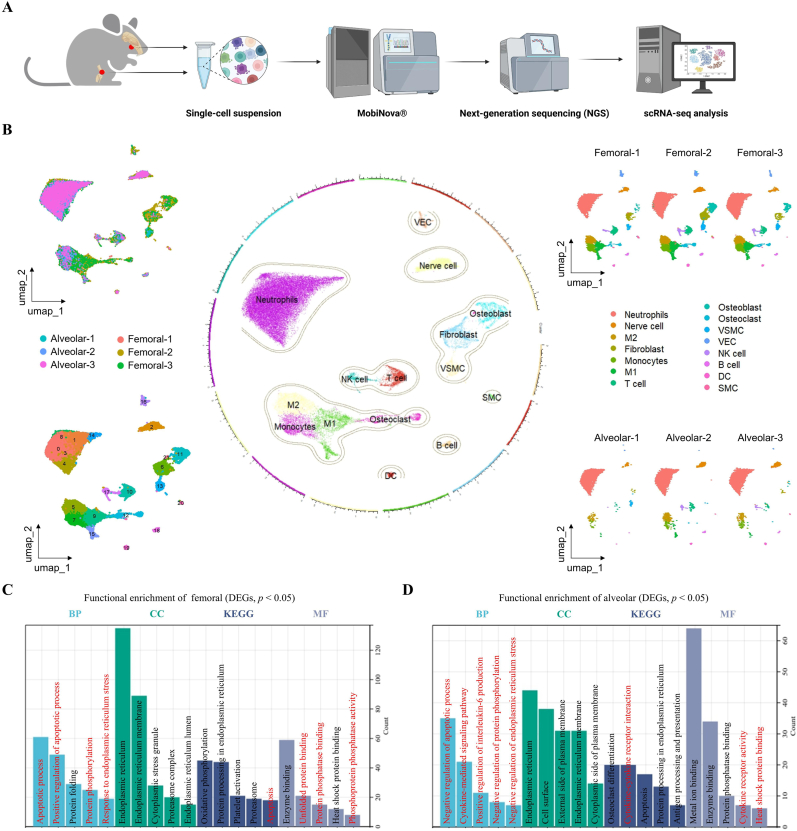
scRNA-seq analysis of early healing tissues in femoral and alveolar bone defects. (**A**) Workflow for scRNA-seq of early defects in rat femoral and alveolar tissues. (**B**) Combined cellular profiles. (**C**, **D**) KEGG and GO enrichment analysis of DEGs.

Reparative tissues were acquired on day 4 to determine the early cellular alterations in femoral and alveolar bone defects, and single-cell suspensions were prepared for scRNA-seq analysis (Fig. 4A). After rigorous quality control employing “Cell Ranger” software and the “Seurat” package, a dataset comprising 34,577 cells, with an average of approximately 1000 feature_RNAs per cell, was achieved (Fig. S4A). Using the expression patterns of canonical cell-type-specific markers, a total of 15 distinct cell populations were identified, encompassing neutrophils (*S100a8*, *S100a9*), nerve cells (*P2rx7*, *Trpc3*), M2 macrophages (*Arg1*, *Il10*), fibroblasts (*Col1a1*, *Col1a2*), monocytes (*Cd14*, *Ccr2*), M1 macrophages (*Cd68*, *Cd86*), T cells (*Cd3g*, *Cd3d)*, osteoblasts (*Alpl*, *Col2a1*), osteoclasts (*Acp5*, *Ctsk*), vascular smooth muscle cells (VSMC; *Cd248*, *Actg2*), vascular endothelial cells (VEC; *Pecam1*, *Cdh5*), natural killer cells (NK cells; *Nkg7*, *Klrg1*), B cells (*Cd79a*, *Cd79b*), dendritic cells (DC; *Flt3*, *Siglech*), and smooth muscle cells (SMC; *Myod1*) (Fig. 4B and Fig. S4B and C). The segmented cellular landscape and proportional stacked plots revealed significant disparities in the cellular composition between the early stages of femoral and alveolar bone defects (Fig. 4B and Fig. S4D).

A cellular localization analysis of the pivotal genes, *Hspa5* and *Il-6*, was conducted from the analyses mentioned above. According to the results, *Hspa5* exhibited ubiquitous expression across diverse cell types, whereas *Il-6* was predominantly and highly expressed in M2 macrophages, with trace amounts in monocytes and VSMC (Fig. S4E). Additionally, intercellular communication analysis revealed extensive interactions between the different cell types (Fig. S5A). To further elucidate the overall functional dynamics during the early repair process of femoral and alveolar bone defects, DEGs (*p* < 0.05) were extracted from the cellular microenvironment of the regenerating tissue and subjected to KEGG and GO enrichment analyses. In the early phase of femoral bone defect, a significant enrichment of processes, such as positive regulation of apoptosis, protein phosphorylation, ERS response, unfolded protein binding, and heat shock protein binding, was observed (Fig. 4C). Conversely, the results revealed notable enrichment of negative regulation of apoptosis, positive regulation of *Il-6* production, negative regulation of ERS, negative regulation of protein phosphorylation, and heat shock protein binding in the early phase of the alveolar bone defect (Fig. 4D). The enrichment results provided further support for the bulk RNA-seq and protein identification findings described above, indicating that similarity of the signaling pathways identified through enrichment.

### 3.4 Regulation of HSPA5 expression by IL-6 in early alveolar bone defects

**Fig. 5:**
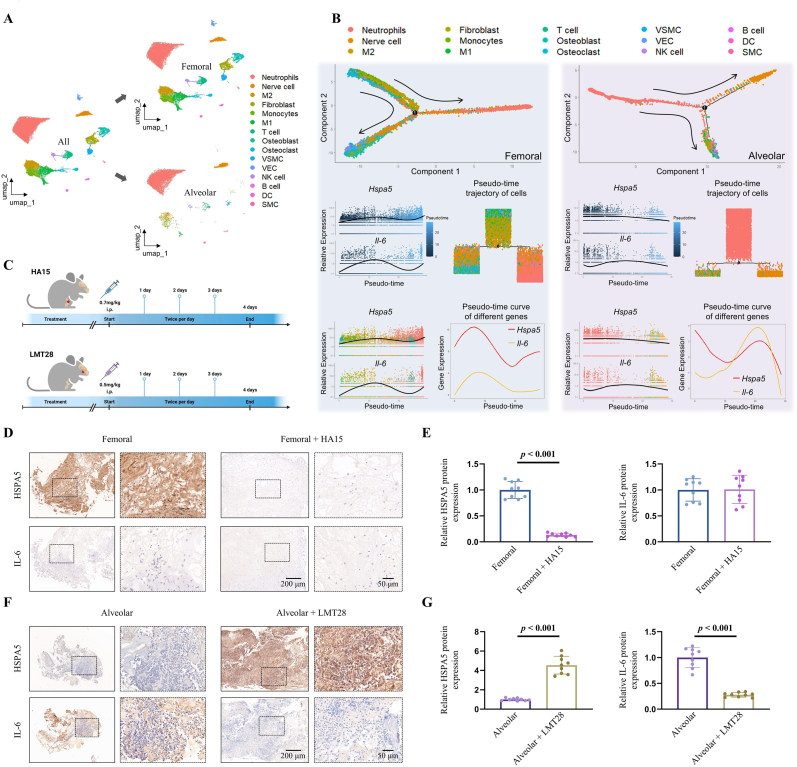
IL-6-mediated regulation of HSPA5 in early bone defect repair tissues. (**A**) Extraction of femoral and alveolar bone cell clusters from whole-cell atlases. (**B**) Pseudo-time series analysis of cellular and gene expression in repair tissues. (**C**) The rat model for femoral and alveolar bone defect treatment using HA15 and LMT28. (**D**, **E**) Immunohistochemical analysis of HSPA5 and IL-6 expression in femoral defects after HA15 treatment. Scale bars: 200 μm and 50 μm. (**F**, **G**) Immunohistochemical analysis in alveolar defects after LMT28 treatment. Scale bars: 200 μm and 50 μm. HA15 inhibits HSPA5; LMT28 inhibits IL-6. Statistical significance was assessed with a *t*-test.

The enrichment results mentioned above suggest a potential regulatory relationship between the key genes *Hspa5* and *Il-6* during the early healing process of bone defects. Based on these findings, grouped extraction of cells from the early stages of femoral and alveolar bone defects was conducted (Fig. 5A). Subsequently, a pseudo-time analysis was employed to map the differentiation trajectories of femoral and alveolar bone-related cells to predict the temporal expression patterns of the key genes. Widespread expression of *Hspa5* was observed across multiple cell types in the early stages of femoral defects, with *Il-6* upregulation primarily at M2 macrophages (Fig. 5B and Fig. S6A). In the temporal expression profile of femoral genes, *Hspa5* exhibited a high early expression peak. At the same time, *Il-6* peaked during the same phase but at a relatively lower expression level (Fig. 5B). In contrast, the cellular expression patterns of *Hspa5* and *Il-6* in the early stages of alveolar bone defects were similar to those in femoral bone. The initial stages exhibited comparable expression of *Hspa5* and *Il-6* in the temporal expression profile of alveolar bone genes (Fig. 5B and Fig. S6B). However, in the middle to late stages, *Il-6* expression rapidly increased, accompanied by *Hspa5* downregulation, and as *Hspa5* declined, *Il-6* also subsequently decreased (Fig. 5B).

An immunocyte analysis was conducted on the bulk RNA-seq results to corroborate the aforementioned analytical findings, revealing a notable increase in macrophage counts in the alveolar bone (Fig. S6C and D). Subsequently, a temporal gene expression analysis was performed at the RNA level on this dataset. The results were consistent with the pseudo-time analysis, demonstrating that *Il-6* was highly expressed in middle-to-late stages of alveolar bone defects, whereas *Hspa5* exhibited high expression primarily in the early-to-middle stages of femoral defects (Fig. S6E).

As shown in Fig. 5C, HSPA5 inhibitor HA15 was administered to the rat femoral bone defect model and IL-6 inhibitor LMT28 to the rat alveolar bone defect model. Tissue samples were collected, and immunohistochemical staining for key gene expression proteins was performed at the early stage of the defect (i.e., day 4). The results indicated that HSPA5 protein was significantly inhibited upon the administration of HA15; however, no significant difference in IL-6 expression was observed compared to the untreated group (Fig. 5D and E). IL-6 was notably suppressed in the early stage of alveolar bone defect treated with LMT28, while HSPA5 expression levels significantly increased (Fig. 5F and G).

Pseudo-temporal observations were conducted for each cell population due to the distinct cellular distribution and proportions in the femoral and alveolar bones. According to the results, immune cells were predominantly present in the early stages of femoral defects. However, functional cells, including fibroblasts, osteoblasts, and vascular cells, became dominant in the later stages, although neutrophils were also observed in the later stages (Fig. S6F). In contrast, most cells emerged in the middle-to-late stages of alveolar bone defects except neutrophils. These findings suggest that the repair process of alveolar bone may be faster than that of femoral bone, which might be attributed to the regulatory role of IL-6 on HSPA5 (Fig. S6F).

### 3.5 Elevated IL-6 in M2 macrophages during alveolar bone healing

**Fig. 6:**
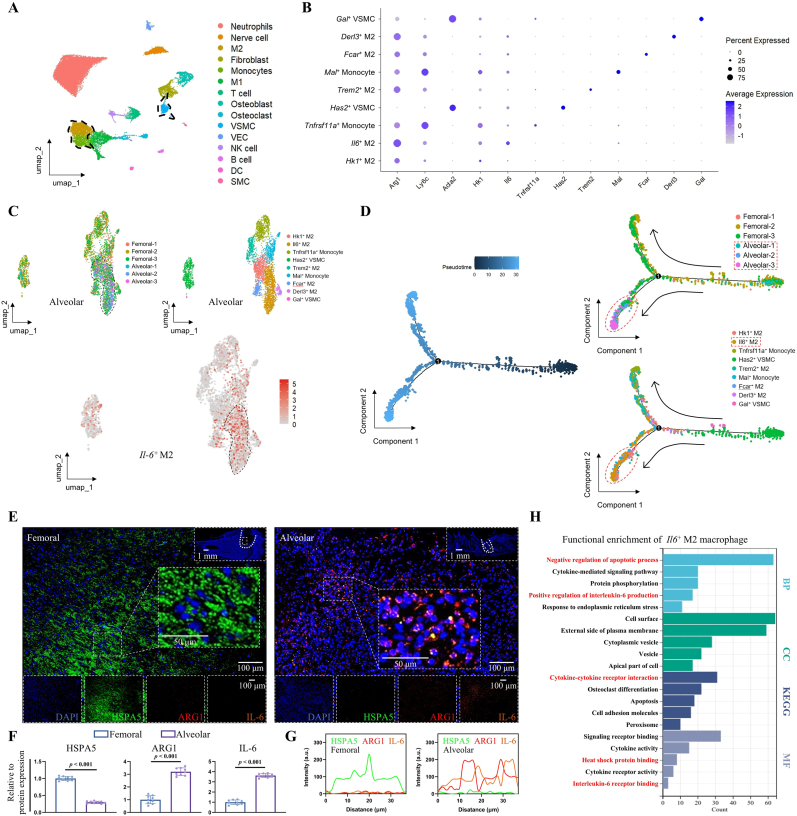
M2 macrophages release IL-6 in alveolar bone defect repair. (**A**) Extraction of *Il-6*-expressing cell populations. (**B**) Identification and classification of subpopulations within *Il-6*-expressing cells. (**C**) Identification of the *Il-6*^+^ M2 cell subpopulation. (**D**) Temporal association between *Il-6*^+^ M2 cell subpopulation and samples. (**E**–**G**) Multiplex immunofluorescence for HSPA5 (GRP78/BiP), M2 macrophages (ARG1), and IL-6, combined with statistical analysis of fluorescence intensity and colocalization analysis. Scale bars: 1 mm, 100 μm and 50 μm. (**H**) KEGG and GO enrichment analysis of DEGs in the *Il-6*^+^ M2 cell subpopulation.

The cellular populations expressing *Il-6* were isolated and segregated into nine distinct sub-clusters: *Hk1*^+^ M2 (*Arg1*, *Hk1*), *Il-6*^+^ M2 (*Arg1*, *Il-6*), *Trem2*^+^ M2 (*Arg1*, *Trem2*), *Fcar* ^+^ M2 (*Arg1*, *Fcar*), *Derl3*^+^ M2 (*Arg1*, *Derl3*), *Tnfrsf11a* ^+^ Monocyte (*Ly6c*, *Tnfrsf11a*), *Mal* ^+^ Monocyte (*Ly6c*, *Mal*), *Has2*^+^ VSMC (*Acta2*, *Has2*), and *Gal* ^+^ VSMC (*Acta2*, *Gal*) (Fig. 6A and B and Fig. S7A). Then, a group-based clustering and IL-6 expression localization analysis was performed on these nine sub-clusters, revealing that *Il-6* was predominantly expressed in early-stage alveolar bone defect samples and localized within the M2 macrophage sub-cluster (Fig. 6C). Furthermore, pseudo-time trajectory analysis of the nine sub-clusters indicated that *Il-6*^+^ M2 macrophages are significantly increased during the early stages of alveolar bone defect healing (Fig. 6D). Validation via multiplex immunofluorescence (mIF) corroborated the aforementioned findings, demonstrating significantly increased HSPA5 expression within femoral granulation tissues at day 4 post-injury, while expression of M2 macrophages (marked by ARG1) and IL-6 was less apparent (Fig. 6E–G). In contrast, alveolar bone exhibited reduced HSPA5 expression but prominent expression of M2 macrophages (marked by ARG1) and elevated IL-6 levels. Notably, IL-6 predominantly localized adjacent to ARG1-positive M2 macrophages within the alveolar bone (Fig. 6E–G).

To further investigate the functional roles of *Il6*^+^ M2 macrophages during the early stages of alveolar bone defects, their DEGs were extracted, and KEGG and GO enrichment analyses were conducted. Notably, pathways such as negative regulation of apoptotic processes, positive regulation of IL-6 production, cytokine-cytokine receptor interaction, heat shock protein binding, and IL-6 receptor binding were significantly enriched (Fig. 6H). These findings are consistent with the enrichment results in our previous analysis of early alveolar bone defects, confirming the crucial function of *Il-6*^+^ M2 macrophages in this early phase.

The above results indicate that IL-6 modulates the protein expression of HSPA5 in various cell types. The *Il-6*^+^ M2 macrophages were isolated and categorized into whole-cell populations to investigate the intercellular interactions (Fig. S8A). Cellular communication analysis revealed that *Il-6*^+^ M2 macrophages interact significantly with other cell types (Fig. S8B and C). Given the significant differences observed in neutrophils during the early stages of femoral and alveolar bone defects in pseudo-time series analysis, neutrophils were isolated and sub-clustered using specific gene markers. Also, seven distinct neutrophil subsets were identified (Fig. S9A–D). Notably, the abundance of *Fhod3*^+^ neutrophils in the early phase of alveolar bone defects was approximately tenfold higher than that in femoral defects (Fig. S9E).

A pseudo-time series analysis was conducted on *Fhod3*^+^ neutrophils and *Il-6*^+^ M2 macrophages, which revealed a dynamic transition from an initial predominance of *Fhod3*^+^ neutrophils, followed by a substantial increase in *Il-6*^+^ M2 macrophages, ultimately culminating in their peak abundance (Fig. S9F). Intercellular communication analysis underscored the interaction between *Fhod3*^+^ neutrophils and *Il-6*^+^ M2 macrophages (Fig. S9G). IF co-localization experiments revealed that, compared to femoral defects, the numbers of *Fhod3*^*+*^ neutrophils and M2 macrophages were significantly higher in alveolar bone defects. Notably, pronounced co-localization of these two cell types was observed during the early stages of alveolar bone defects (Fig. S9H–J). This finding underscores the specific spatial-temporal interaction between *Fhod3*^+^ neutrophils and M2 macrophages in the early phases of alveolar bone repair.

### 3.6 IL-6-mediated inhibition of HSPA5-induced apoptosis in ERS

A comprehensive analysis was conducted, integrating the enrichment outcomes from bulk RNA-seq, protein identification, and scRNA-seq, revealing the ERS response's predominance and associated apoptotic processes during the early stages of femoral defects (Fig. S10A). In contrast, although endoplasmic reticulum stress and apoptotic processes are also present in the initial phase of alveolar bone defects, they are overshadowed by the adverse regulatory effects mediated by IL-6 (Fig. S10B).

**Fig. 7:**
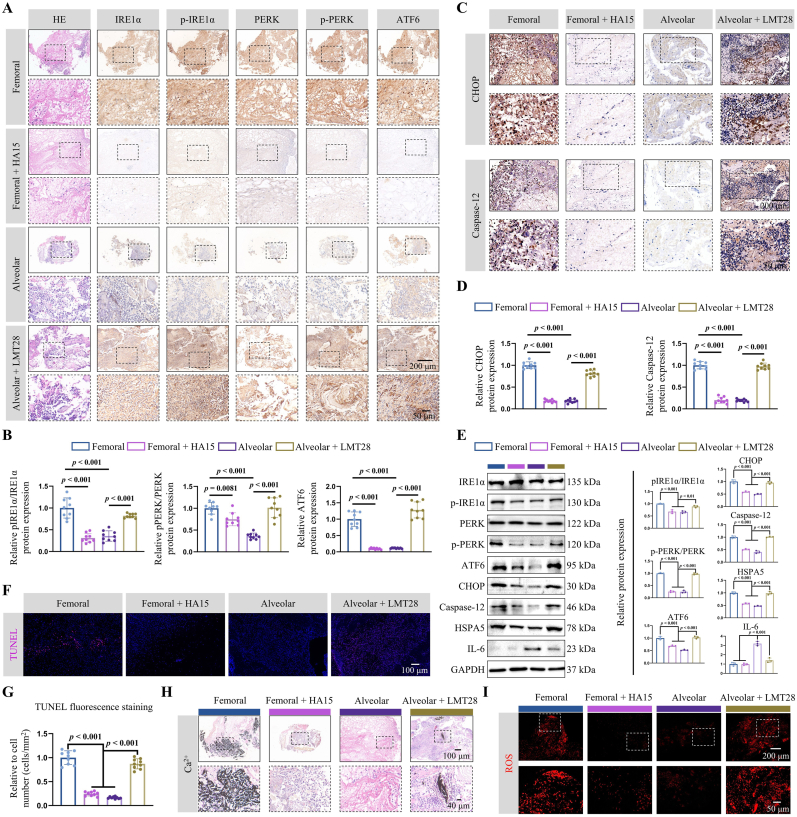
IL-6 modulates HSPA5 to mitigate endoplasmic reticulum stress (ERS)-related apoptosis in early bone defects. (**A**, **B**) Expression and quantitative analysis of ERS-related proteins following HA15 and LMT28 treatments. Scale bars: 200 μm and 50 μm. (**C**, **D**) Quantitative analysis of apoptosis-related proteins CHOP and caspase-12 after treatment. Scale bars: 200 μm and 50 μm. (**E**) Expression levels of ERS and apoptosis-related proteins were analyzed by Western blot, and statistical analysis was conducted on the results. (**F**, **G**) Quantitative terminal deoxynucleotidyl transferase dUTP nick-end labeling (TUNEL) staining and statistical analysis of apoptosis in femoral and alveolar bone defects. Scale bars: 100 μm. (**H**) Ca^2+^ histopathological staining. Scale bars: 100 μm and 40 μm. (**I**) Detection of reactive oxygen species in repair tissue following HA15 and LMT28 treatment. Scale bars: 200 μm and 50 μm. Statistical significance was determined using one-way ANOVA.

To validate the combined analysis's findings, IHC staining was performed to examine the expression of ERS-related proteins and apoptotic proteins in the nascent tissues of femoral and alveolar bone defects on 4 days. The results revealed significantly lower expression levels of ERS-related proteins, including p-IREα/IREα, p-PERK/PERK, and ATF6 (Fig. 7A and B), and apoptotic proteins such as CHOP and Caspase-12 (Fig. 7C and D), compared to femoral bone, in the early stages of alveolar bone defects. Treatment with HA15 converged the expression patterns of ERS and apoptosis-related proteins in early femoral defects towards those observed in the alveolar bone (Fig. 7A–D). In contrast, the application of LMT28 markedly upregulated these proteins in the early alveolar bone defects (Fig. 7A–D). Furthermore, the IF staining of ERS-related proteins was consistent with the IHC findings, confirming lower expression levels during the early stages of alveolar bone defects. Notably, LMT28 treatment reversed this trend, significantly upregulating ERS-related proteins (Fig. S11A and B). The results of the Western blot were further analyzed to verify ERS and apoptosis-related proteins in early-stage (4 days) repair tissues of femoral and alveolar bone defects. The results demonstrated a trend consistent with IHC findings: HA15 treatment suppressed the expression of ERS and apoptosis-related proteins in the femur, whereas LMT28 application enhanced their expression (Fig. 7E). Similarly, to mitigate potential off-target effects of single inhibitors, additional inhibitors were used to validate the reliability of the observations. Rats were treated with HM03 (an HSPA5 protein inhibitor) and SC144 (an IL-6 protein inhibitor) following an identical protocol. Granulation tissues from bone defects at 4 days post-injury were then harvested for ERS and apoptosis-related protein detection. Results aligned with prior data, showing that HM03 treatment suppressed ERS and apoptosis-related protein expression in the femur, while SC144 application enhanced their expression (Fig. S11C and D).

The TUNEL staining was utilized to detect 3′-OH ends generated by DNA fragmentation in apoptotic cells within the repair tissues of femoral and alveolar bone defects at the early stage (4 days). The results revealed increased apoptotic cell numbers in both the alveolar bone and femur following LMT28 treatment. In contrast, HA15 administration significantly reduced apoptotic cells in the femur, approximating the levels observed in alveolar bone (Fig. 7F and G). The levels of Ca^2+^ and ROS associated with early-stage ERS in femoral and alveolar bone defects were examined. Notably, the initial stages of alveolar bone defects were associated with a marked decrease in both Ca^2+^ and ROS levels compared to femoral defects. Subsequent application of HA15 reversed the trends in Ca^2+^ and ROS content observed in early-stage femoral defects. Conversely, LMT28 administration significantly upregulated Ca^2+^ and ROS levels in the early phases of alveolar bone defects (Fig. 7H, I and Fig. S11E and F). These findings reinforce the phenomenon mentioned above and elucidate the regulatory role of IL-6 in modulating HSPA5-mediated ERS and apoptosis processes.

### 3.7 Initial healing in femoral defects accelerated by local IL-6 administration

**Fig. 8:**
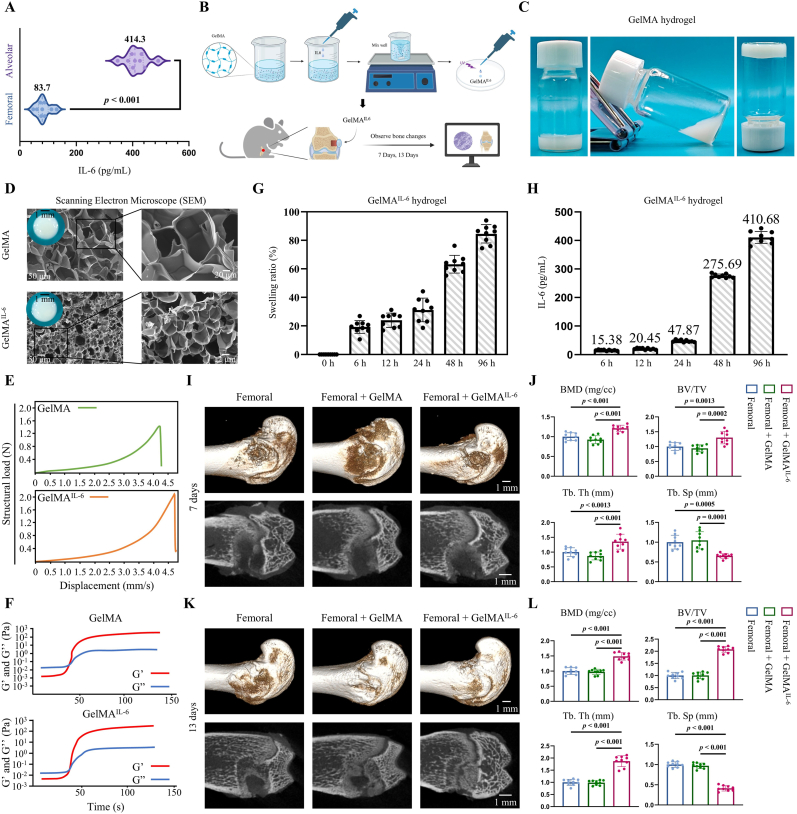
GelMA^IL−6^ hydrogel promotes early repair of femoral defects. (A) ELISA measurement of IL-6 concentration in early-stage (4 days) defects. (B) Schematic representation of GelMA^IL−6^ hydrogel preparation and therapeutic mode. (C) Photocrosslinking enables phase transition of liquid hydrogels into solid hydrogels while conferring shape-editing capability during the curing process. (D) General view and SEM images of GelMA hydrogel and GelMA^IL−6^ hydrogel. Scale bars: 1 mm, 50 μm, and 20 μm. (E, F) Characterization of compressive modulus and rheological properties assessment for GelMA hydrogel and GelMA^IL−6^ hydrogel. (G) Swelling ratio of GelMA^IL−6^. (H) ELISA analysis of sequential IL-6 release from GelMA^IL−6^ hydrogel. (I–L) μCT analysis and quantification of femoral defects on days 7 and 13 after GelMA^IL−6^ application. Scale bar: 1 mm. Statistical analysis was performed with one-way ANOVA.

It is hypothesized that IL-6 is a pivotal factor in the early stages of bone defect repair, based on the analyses and experimental outcomes mentioned above. IL-6 concentrations in the early phases of femoral and alveolar bone defects were quantified using the ELISA method. Notably, the IL-6 concentration in femoral defects on day 4 was 83.7 ± 23.8 pg/mL. In contrast, its concentration was 414.3 ± 38.1 pg/mL in alveolar bone defects (Fig. 8A). IL-6 was encapsulated within GelMA hydrogels and applied to femoral defects to evaluate their osteogenic potential, mimicking the elevated IL-6 levels observed in early alveolar bone defects (Fig. 8B). The fabricated GelMA hydrogel exhibited injectability and could be photochemically crosslinked into various configurations (Fig. 8C). Both resulting GelMA and GelMA^IL−6^ hydrogel implants displayed a translucent appearance. The SEM images revealed a densely interconnected porous network within the microstructure of both hydrogels (Fig. 8D). Notably, the porous network of GelMA^IL−6^ exhibited significantly greater compactness compared to that of GelMA. Compressive modulus testing revealed a marginally higher structural load-bearing capacity in GelMA^IL−6^ hydrogels compared to GelMA ones, probably attributable to increased crosslinking density following protein incorporation (Fig. 8E). This demonstrates that both hydrogels possess significant stress-bearing capabilities. Furthermore, rheological analysis assessment showed no significant differences between GelMA and GelMA^IL−6^ hydrogels (Fig. 8F).

The swelling behavior of GelMA^IL−6^ hydrogel was evaluated. The results demonstrated that the swelling ratio progressively increased within 0–24 h, and then surged to 80 % during the 48–96 h period (Fig. 8G). Concurrently, IL-6 release kinetics during hydrogel hydrolysis were quantified. Similarly, IL-6 levels exhibited a rapid surge between 24 and 48 h, reaching a concentration of 410.7 pg/mL by 96 h (Fig. 8H). This hydrogel system, featuring matched swelling ratio and IL-6 release kinetics, closely mimicked the physiological IL-6 concentration (414.3 pg/mL) previously detected in alveolar bone defect tissues, effectively recapitulating *in vivo* IL-6 dynamics. Additionally, culture media conditioned with GelMA^IL−6^ for 96 h were used to stimulate cells *in vitro* (Fig. S12A). Live/dead staining after 96 h of stimulation indicated no significant cytotoxicity, demonstrating the biocompatibility of GelMA^IL−6^ under these conditions (Fig. S12B and C).

Radiographic observations were performed on femoral defects treated with local application of IL-6 for 7 and 13 days, respectively. The μCT reconstructions indicated that localized administration of IL-6 promoted new bone formation in the femoral defects compared to both the untreated and GelMA-only groups (Fig. 8I–L). Quantitative μCT analyses demonstrated increased BMD, BV/TV, and Tb.Th, along with a decrease in Tb.Sp in the GelMA^IL−6^ group (Fig. 8I–L). Notably, after 7 days of treatment, the extent of bone healing was slightly higher in the GelMA^IL−6^ group than in the untreated and GelMA-only groups (Fig. 8I and J). However, by day 13, the GelMA^IL−6^ treatment group exhibited significantly greater bone healing (Fig. 8K and L).

### 3.8 Mitigation of ERS and apoptosis by IL-6 in femoral defects

**Fig. 9:**
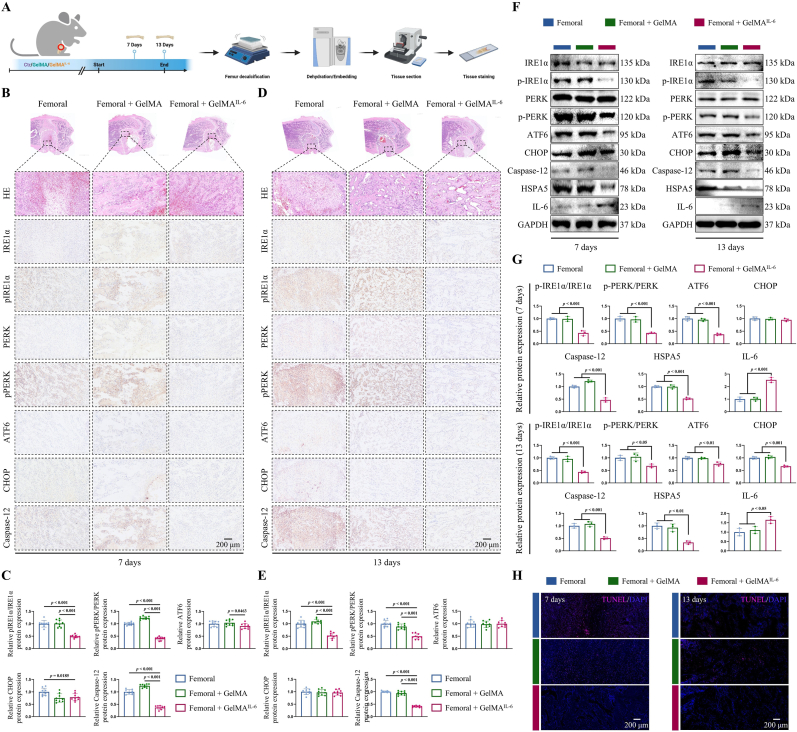
GelMA^IL−6^ hydrogel facilitates bone healing by mitigating ERS and apoptosis. (**A**) Schematic representation of histopathological assessment of femoral tissue after GelMA^IL−6^ therapy. (**B**, **C**) Histological analysis on day 7 after treatment, including HE and IHC results, with quantitative analysis. Scale bar: 200 μm. (**D**, **E**) Histological examination on day 13 after treatment, including HE and IHC results, with quantitative analysis. Scale bar: 200 μm. (**F**, **G**) Expression levels and quantitative analysis of endoplasmic reticulum stress (ERS)-related and apoptosis-related protein expression in femoral defect tissues at 7 and 13 days post GelMA^IL−6^ treatment administration. (**H**) Terminal deoxynucleotidyl transferase dUTP nick-end labeling (TUNEL) staining of apoptotic cells in femoral defect tissues at 7 and 13 days post-treatment with GelMA^IL−6^. Scale bar: 200 μm. Statistical significance was determined using one-way ANOVA.

Femoral bone tissues harvested from animals treated with GelMA^IL−6^ on days 7 and 13 were decalcified, dehydrated, embedded, and sectioned to determine histopathological changes (Fig. 9A). According to the results, 7 days after treatment, the GelMA^IL−6^ group exhibited significant downregulation of ERS-related proteins (pIRE1α/IRE1α and pPERK/PERK) compared to the control group and the GelMA group (Fig. 9B and C). Additionally, the apoptotic protein Caspase-12 was notably downregulated. Consistent with the findings on day 7, on day 13, IL-6 treatment significantly downregulated the ERS-related proteins (pIRE1α/IRE1α and pPERK/PERK) and the apoptotic protein Caspase-12 (Fig. 9D and E). IL-6 treatment decreased the area of bone defects. Notably, in the 7- and 13-day femoral defects, the ATF6 and CHOP proteins exhibited slight downregulation in the IL-6-treated group.

The ROS levels were quantified in femoral defects on days 7 and 13 post-injury. At day 7, both control and GelMA-treated groups exhibited elevated ROS concentrations compared to baseline. Notably, IL-6 administration significantly attenuated ROS accumulation in bone defects at this time point (*p* < 0.05) (Fig. S13A and B). Similar observations were found at 13 days after creating the defects, with IL-6 treatment significantly downregulating ROS levels (Fig. S13C and D). Western blot analysis of ERS and apoptosis-related protein expression at 7 and 13 days revealed a trend comparable to the IHC staining results (Fig. 9F and G). Subsequently, TUNEL staining demonstrated that, compared with both the untreated group and the GelMA hydrogel group, GelMA^IL−6^ treatment significantly reduced the number of apoptotic cells within the femoral defect tissues (Fig. 9H and Fig. S13E). These findings suggest that the release of IL-6 can mitigate ERS and its associated apoptotic processes during the early stages of bone defect repair, promoting bone tissue healing.

## 4 Discussion

Alveolar and femoral bone defect healing is modulated by vascularization and mechanical stress. Augmented vascularization in alveolar bone contributes to its superior reparative capacity, whereas mechanical stress stimulation following osseous injury enhances osteogenic repair in femoral bone [34,35]. Nevertheless, during the initial phase of bone defect healing—when vascular networks remain unestablished and mechanical stimuli are suboptimal—the precise reparative mechanisms persist as elusive.

This study examined the histological changes in the healing tissues of femoral and alveolar bones at different stages of injury repair, i.e., 2-, 4-, 7-, and 13-day intervals [6]. Two days following bone defect formation, the femoral defect site was predominantly occupied by blood cells. In contrast, the alveolar bone exhibited the emergence of collagen fiber-like tissue within its defect region. On day 4, a minor quantity of collagen fiber-like tissue had appeared in the femur. Concurrently, the alveolar bone demonstrated denser collagen fiber-like tissue accompanied by substantial lymphocyte infiltration. As the healing process advanced, ossification occurred in the alveolar bone, whereas femoral ossification progressed at a comparatively slower rate. These findings suggest heterogeneity in the healing processes and mechanisms between the alveolar and femoral bones after injury, highlighting the potentially pivotal role of early stages. Significant lymphocyte infiltration was observed in the alveolar bone at day 4 post-injury. This phenomenon may be associated with IL-6-mediated suppression of ERS. Following ERS induction, cells exhibit stimulated sphingosine synthesis. This leads to sphingosine accumulation within the endoplasmic reticulum, which reduces membrane fluidity and consequently impairs cellular migration [36]. Conversely, IL-6 inhibits ERS occurrence in alveolar bone tissues. This suppression likely contributes to accelerated cellular migration that manifests histologically as lymphocyte infiltration.

A joint analysis integrating bulk RNA-seq, protein identification, and scRNA-seq was conducted to further elucidate the underlying mechanisms governing the early healing processes of femoral and alveolar bones. The results indicated the presence of ERS during the early stages of femoral defect repair, with excessive ERS further triggering apoptosis and inhibiting collagen fiber and new bone formation. In contrast, M2 macrophages in the alveolar bone secrete an optimal amount of IL-6, maintaining ERS homeostasis during the repair process and suppressing apoptosis. Previous studies have demonstrated that ERS homeostasis promotes new bone formation, collagen fiber proliferation, and bone density enhancement [37]. In contrast, ERS imbalance and the subsequent apoptosis process can lead to inflammation and retard bone healing [38]. This is consistent with our findings, indicating that IL-6 regulates ERS during the early stages of alveolar bone defects and facilitates bone regeneration.

Cellular localization revealed high expression of *Hspa5* in various cells during the early stage of bone defects, as confirmed by histological findings. Subsequently, it was observed that high HSPA5 expression mediated extensive manifestation of ERS and apoptosis in the early phase of femoral defects, which resembles a cytokine storm, where immune cells release a surge of inflammatory factors in response to foreign invaders or to clear abnormalities [39]. During this process, once the immune system prevails, it reduces cytokine release to maintain homeostasis. However, when the immune system becomes overly activated, excess cytokine production leads to a cytokine storm. These excess cytokines evade the immune control and attack all cells in the body, inducing systemic inflammation, organ failure, and even death [40]. In the early stages of bone defects, ERS shows a similar pattern, with widespread and active ERS detected in early femoral defects, culminating in apoptosis. In contrast, alveolar bone defects are associated with more stable ERS in the presence of IL-6, seldom leading to severe apoptosis. This stability may reflect the body's stress response to sudden events during the early stage of bone defects. It is proposed that this may represent an “excessive endoplasmic reticulum stress” (i.e., ERS storm) phenomenon in the early stages of bone defects with low IL-6 levels, analogous to a cytokine storm.

The scRNA-seq analysis revealed significant intercellular heterogeneity during the early stages of femoral and alveolar bone regeneration, manifested primarily by differences in cell differentiation pathways and cellular subtypes. Pseudo-time series analysis revealed that *Il-6* expression in the femur was only slightly elevated at the initial stage, with overall insignificant changes. In contrast, *Il-6* expression in alveolar bone gradually increased over time, accompanied by *Hspa5* downregulation, suggesting that specific cells secrete IL-6 during the early stages of alveolar bone defects, which are less abundant in the femur. Additionally, IL-6 can regulate HSPA5 and its downstream mechanisms. According to the existing evidence, the initial inflammatory phase and its subsequent resolution create a favorable environment for tissue repair and bone regeneration [41,42]. IL-6 inhibition during the early stages of bone defects decreases inflammation; however, the recruitment of immune cells and bone regeneration are also suppressed, delaying fracture healing [8,43]. In the process of bone regeneration, signaling pathways and cytokines beyond IL-6 and HSPA5 play critical roles. For instance, the Wnt signaling pathway promotes bone formation by regulating osteoblast differentiation and function [44]. Members of the bone morphogenetic protein (BMP) family, such as BMP-9, are potent factors for inducing the osteogenic differentiation of bone marrow mesenchymal stem cells and are widely applied in bone tissue engineering [45]. Furthermore, the fibroblast growth factor signaling pathway is also significantly involved in the regulation of osteoblast proliferation and differentiation [46]. These signaling pathways, together with cytokines like IL-6, could form a complex regulatory network, thus orchestrating the progression of bone regeneration.

At the cellular subtype level, highly expressed *Il-6* were isolated and re-clustered, revealing the presence of a unique M2-like cell subpopulation during the early stages of alveolar bone repair. This subpopulation serves as the primary source of IL-6 secretion. While IL-6 is commonly associated with proinflammatory cells, the correlation between M2-like macrophages and IL-6 production supports our findings [47]. IL-6 is a crucial inflammatory cytokine in bone healing processes, and excessive inflammation can inhibit new bone and angiogenesis [48]. However, this inflammatory response is not absolute [49]. Furthermore, M2 macrophage-derived extracellular vesicles reduce neuronal apoptosis and improve functional recovery in spinal cord injury [50]. This aligns with our bone defect findings: M2 macrophages suppress apoptosis during early repair, promoting osteogenesis. M1 macrophages drive early inflammation during fracture healing. M2 macrophages help bone repair by releasing cytokines and osteogenic factors that recruit osteoblasts [51]. Furthermore, exosomes derived from M2 macrophages can induce osteogenic differentiation of pluripotent stem cells, further substantiating the pivotal role of M2 macrophages in bone tissue regeneration [52].

An analysis of the upstream regulatory cells of *Il-6*^+^ M2 macrophages revealed that *Fhod3*^+^ neutrophils exhibit specificity towards different bone defect types, predominantly appearing during the early stages of alveolar bone defects. This specificity suggests that *Fhod3*^+^ neutrophils may be regulatory cells triggering endogenous recovery during early bone defect repair. Neutrophils can promote the differentiation of mesenchymal stem cells into osteoblasts by activating BMP and TGF–β signaling pathways; additionally, neutrophil extracellular traps can accelerate ectopic bone formation [53]. Furthermore, *p21*^+^ neutrophils rapidly emerge after bone injury and inhibit aging-related features in fracture healing when their genes are depleted, accelerating the healing process [54]. Therefore, the neutrophil subset differentiation and a comprehensive regulatory network are crucial to clarifying the intrinsic healing mechanisms. In this context, a specific neutrophil subset was identified as being present during the early stages of alveolar bone defect repair, with a possible pivotal role in regulating downstream IL-6 secretion and homeostasis.

The repair processes of alveolar and femoral bone defects are modulated by their local microenvironments. Our study revealed significantly elevated IL-6 expression and enhanced M2 macrophage recruitment in alveolar defects compared to femoral defects, consistent with the heightened immunoreactivity of the alveolar microenvironment [55]. The depletion of specific M2 macrophage subsets disrupts bone healing, while their immunomodulatory functions actively potentiate the reparative cascade [56]. Furthermore, within the bone marrow microenvironment, competitive cytokine milieus (e.g., TGF-β, IL-10, IL-1β) may drive M1-to-M2 macrophage phenotypic switching, thereby inducing immunoreactivity dysregulation that undermines local inflammatory orchestration and impedes reparative progression [57]. These findings underscore that deciphering microenvironmental heterogeneity is paramount for developing targeted therapeutic strategies.

IL-6 is conventionally regarded as a classic pro-inflammatory cytokine, primarily produced by M1 macrophages, and plays a pivotal role in numerous inflammation-associated diseases [58]. However, the observation of delayed wound healing in patients administered with anti-IL-6 antibodies indicates a dual role for IL-6 in tissue repair processes [59]. Our study reveals that within the specific microenvironment of early alveolar bone defect repair, IL-6 exhibits functional characteristics distinct from its traditional perception. During the initial healing phase of alveolar bone, M2 macrophages express substantial levels of IL-6. Critically, in this context, IL-6 does not exert pro-inflammatory effects. Instead, it functions by modulating HSPA5 expression and the ERS response, effectively mitigating ER stress-induced apoptosis. This mechanism is essential for maintaining cellular homeostasis during the early stages of bone repair, thereby creating favorable conditions for the survival of osteoblasts and the normal deposition of bone matrix. Consequently, IL-6 promotes the progression of alveolar bone regeneration. This discovery not only expands our understanding of the functional complexity of IL-6 but also provides a novel scientific rationale and theoretical foundation for optimizing the design of bone-repair biomaterials and developing targeted therapeutic strategies for bone healing disorders.

GelMA, a photocrosslinkable hydrogel, has gained widespread application in the fields of tissue engineering and drug delivery owing to its excellent biocompatibility, tunable physicochemical properties, favorable cellular interactions, and capacity for controlled drug release [60]. Cytokine-loaded GelMA hydrogels have been used to induce osteogenic differentiation in clinical translational research [61,62]. Song et al. engineered a multifunctional hydrogel therapy by integrating PP5 NMs, the pro-osteogenic protein rhBMP9, and a hydrogel-forming thermo-responsive material, which can regulate the pro-inflammatory/oxidative microenvironment and exhibits excellent bone regeneration capability in bone repair [63]. The GelMA hydrogels were synthesized and loaded with an appropriate concentration of IL-6 to validate our analytical findings and provide potential therapeutic strategies for bone healing. The application of GelMA^IL−6^ hydrogels for sustained release of IL-6 in treating femoral defects significantly accelerated healing, with substantial new bone reconstruction observed at the defect site by day 13 after treatment. This phenomenon suggests that applying IL-6 during the early stages of bone defects can effectively maintain the excessive stress and inflammatory responses after injury at a relatively moderate level, facilitating healing and regeneration. Notably, histological staining at day 13 post-GelMA^IL6^ treatment revealed emergent cartilaginous tissue. This observation likely reflects an active phase of endochondral ossification during early bone healing—a critical transitional stage in physiological repair. The cartilaginous template formed through this process will be progressively replaced by mature bone tissue in subsequent remodeling [64,65]. In addition, biodegradable metals such as magnesium-based and zinc-based alloys hold great promise for clinical translation due to their moderate degradation rates and superior mechanical properties [[66], [67], [68]]. Based on current research, the development of surface-modified bone repair materials loaded with ERS inhibitors or small-molecule targeted drugs offers multiple possibilities for clinical applications in bone regeneration.

Temporal differences may exist in the early ERS and its apoptotic processes in femoral defects [37,69]. Our results indicated significantly increased expression of pIRE1α/IRE1α, pPERK/PERK, and Caspase-12 on days 7 and 13 after inducing defects in the control and GelMA groups, with IL-6 capable of reversing this phenomenon. However, no significant changes in the expression of ATF6 and CHOP proteins were observed on days 7 and 13. Notably, a marked elevation in ATF6 and CHOP protein levels was observed on day 4. The stage-specific differences in ATF6 and CHOP protein expression may be attributed to the modulation of ERS by cellular homeostasis following femoral defects, a process that occurs more gradually than the direct regulation of IL-6 in the alveolar bone. Our findings also suggest this notion, as the expression of ATF6 and CHOP proteins became more stabilized on day 13 compared to day 7.

Admittedly, the experimental approaches in this study have certain limitations. For instance, some of the experimental methods in this study can be further improved. Further research can focus on the genetic editing of *Fhod3*^+^ cells to elucidate their role in regulating downstream cell differentiation. Furthermore, to address the potential off-target effects of inhibitors, conditional gene-editing mouse models—such as bone tissue-specific IL-6 knockout mice and HSPA5 overexpression mice—should be developed. Alternatively, CRISPR-Cas9-mediated *in vivo* gene editing technology could be employed to validate our hypotheses. Applying spatial transcriptomics, flow cytometry, and proteomics to observe the spatial localization and interactions of IL-6 and HSPA5 during the early bone repair phase will further clarify their regulatory relationships. Furthermore, this study employed single-omics analysis with correlative validation—a methodology effective in mitigating analytical noise inherent to multi-omics integration while guiding biomaterial design.

Considering the future direction, the improvement in the femoral bone healing process by IL-6 indicates that this effect is not tissue-specific, offering potential guidance for subsequent clinical translation. In fact, species-specific variations, particularly potential heterogeneity in macrophage polarization and neutrophil function between rats and humans, may constrain the clinical translatability of animal models in immune microenvironment research [70]. Consequently, further validation using human-derived specimens or *in vitro* models is warranted. Further in-depth research and validation are necessary for the early bone healing effects of IL-6, particularly regarding whether the tissue specificity of its secreting cells is regulated by alveolar bone-specific environments, such as abundant blood supply and externally interacting wounds [71]. The intricate microenvironment formed by these factors may also be an essential foundation for IL-6 to exert its effects.

## 5 Conclusion

This study presents the “multi-omics informed hydrogel design” as a promising strategy. The mechanisms behind the heterogeneous healing processes in alveolar and femoral defects were elucidated using multi-omics techniques. It was observed that femoral bone healing in its early stages involves upregulated HSPA5-induced ERS and apoptosis, which hinder local stability and delay repair. In contrast, alveolar bone defects observe a rapid emergence of *Fhod3*^*+*^ neutrophils that regulate IL-6 secretion by *Il-6*^+^ M2 macrophages, countering HSPA5-induced ERS and apoptosis, thus preserving local stability and facilitating rapid recovery. Leveraging these insights, a gelatin-based porous hydrogel was developed and optimized for localized IL-6 delivery in femoral defects, resulting in significant improvement of bone repair by modulating ERS and inflammatory responses. This multi-omics approach enhances our understanding of bone repair mechanisms, enabling biology-driven material design and advancing the development of tailored materials for precise regenerative medicine.

## CRediT authorship contribution statement

**Jiannan Zhou:** Writing – original draft, Visualization, Validation, Software, Resources, Methodology, Investigation, Formal analysis, Data curation. **Jingtao Dai:** Writing – review & editing, Supervision, Project administration, Methodology, Conceptualization. **Shixian Hu:** Supervision, Methodology, Formal analysis. **Cancan Qi:** Methodology, Investigation. **Jiahao Chen:** Methodology, Investigation. **Wentai Zhang:** Supervision, Methodology, Investigation. **Dorothea Alexander:** Writing – review & editing, Methodology. **An Li:** Writing – review & editing, Methodology. **Yin Xiao:** Writing – review & editing, Resources, Funding acquisition, Conceptualization. **Ping Li:** Writing – review & editing, Writing – original draft, Validation, Resources, Methodology, Funding acquisition, Conceptualization.

## Ethics approval and consent to participate

The animal research involved in this work was approved by the Animal Ethics Committee of Guangzhou Medical University (Ethics Approval Number: GY2023-721) on December 15, 2023.

## Data availability statement

The datasets used and/or analyzed during the current study are available from the corresponding author on reasonable request.

## Funding statement

This work was supported by the 10.13039/501100001809National Natural Science Foundation of China (82301134), 10.13039/501100021171Guangdong Basic and Applied Basic Research Foundation (2021A1515111140, 2021B1515120059 and 2022A1515110379), and Science and Technology Projects in Guangzhou (202102080148).

## Declaration of competing interest

The authors declare that they have no known competing financial interests or personal relationships that could have appeared to influence the work reported in this paper.
