# Supplementary material for: Multi-omics-informed hydrogel design: modulating IL-6 to reduce endoplasmic reticulum stress in bone regeneration
Source: Bioact Mater. 2025 Oct 15;56:95–114. doi: 10.1016/j.bioactmat.2025.09.005 (PMC12553026; doi:10.1016/j.bioactmat.2025.09.005)
Supplement: Multimedia component 1 [file mmc1.docx]

**Multi-omics-informed hydrogel design: modulating IL-6 to reduce endoplasmic reticulum stress in bone regeneration**


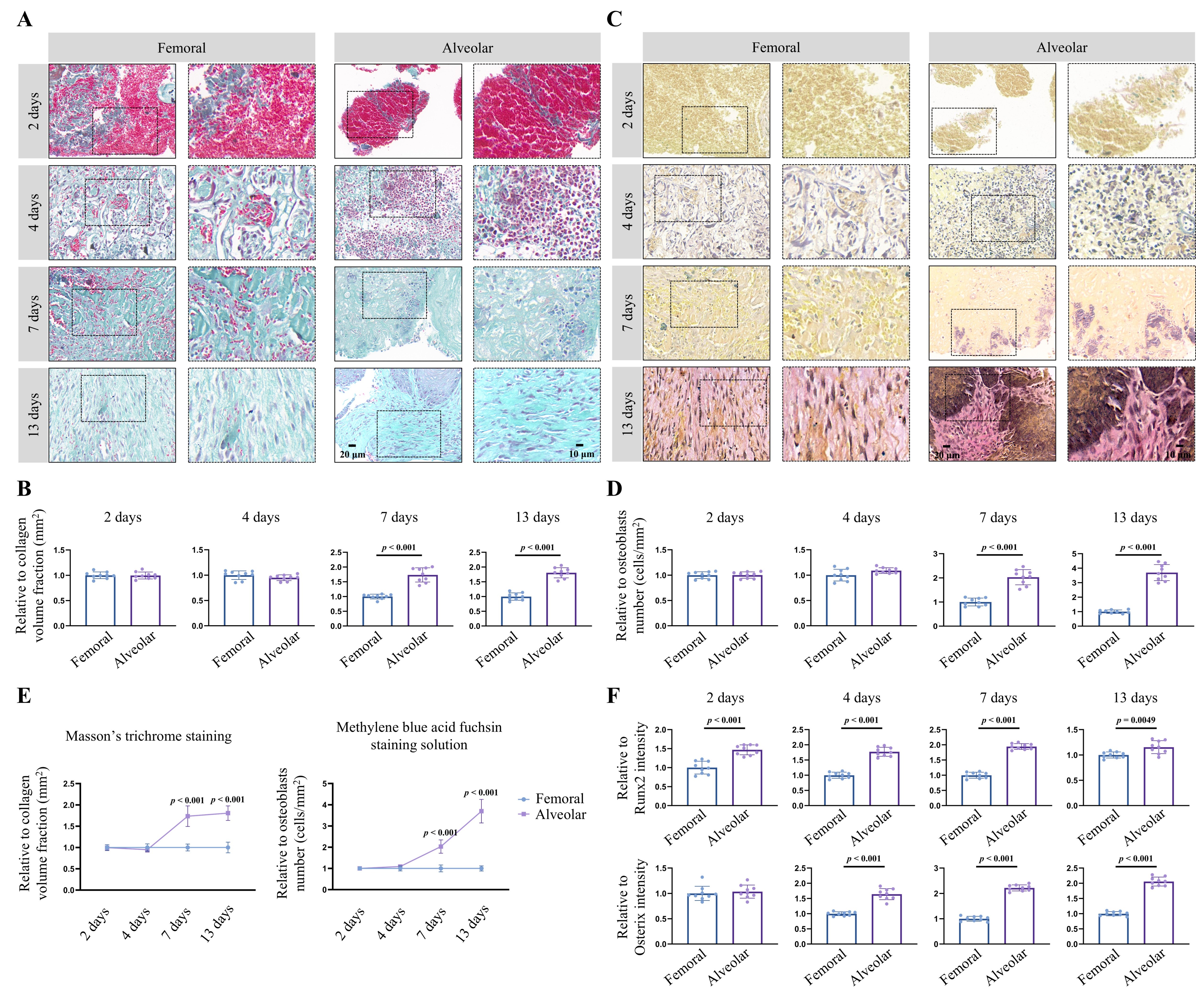


**Fig. S1** Early stage of alveolar bone regeneration exhibits a relatively swift progression. (**A**, **B**) Quantitative analysis of Masson staining results and collagen volume fraction in the early repair tissues of femoral and alveolar bone defects at different time points. Scale bars, 20 μm and 10 μm. (**C**, **D**) Statistical analysis of methylene blue-acid fuchsin staining results and osteoblast counts in the early repair tissues of femoral and alveolar bone defects at various time points. Scale bars, 20 μm and 10 μm. (**E**) Histological assessments of early repair tissues in femoral and alveolar bone defects at various time points, including Masson's trichrome staining and methylene blue-acid fuchsin staining solution. (**F**) Statistical analysis of Runx2 (Runt-related transcription factor) and Osterix protein expression via immunofluorescence staining in early repair tissues of femoral and alveolar bone defects at varying time intervals. Statistical significance was determined using t-test.


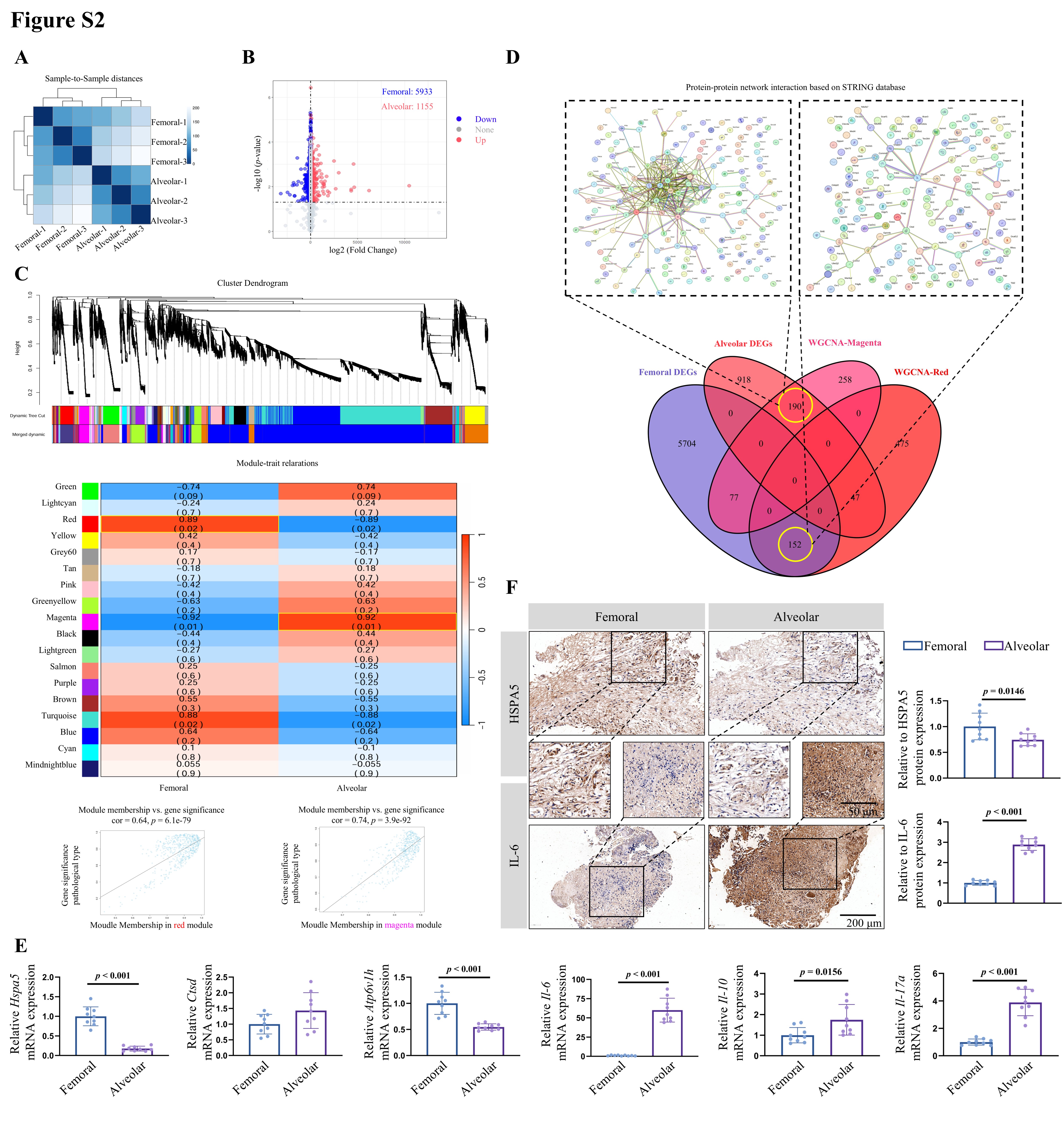


**Fig. S2** Bulk RNA-seq of early repair tissues in femoral and alveolar bone defects. (**A**) Sample clustering and PCA dimensionality reduction in Bulk RNA-seq analysis. (**B**) Identification of differentially expressed genes (DEGs) in early repair of femoral and alveolar bone defects. (**C**) Weighted gene co-expression network analysis (WGCNA) for screening early-stage relevant modules in femoral and alveolar bone defects. (**D**) Venn diagram of DEGs and WGCNA modules, and their protein-protein interaction analysis based on String-DB (https://cn.string-db.org/). (**E**) RT-qPCR was used to detect the RNA expression levels of key genes in early stages of femoral and alveolar bone defects. (**F**) Statistical analysis and immunohistochemical (IHC) detection of HSPA5 (GRP78/BiP) and IL-6 protein expression in callus tissues of femoral and alveolar bone defects at postoperative day 4. Statistical significance was determined using t-test.


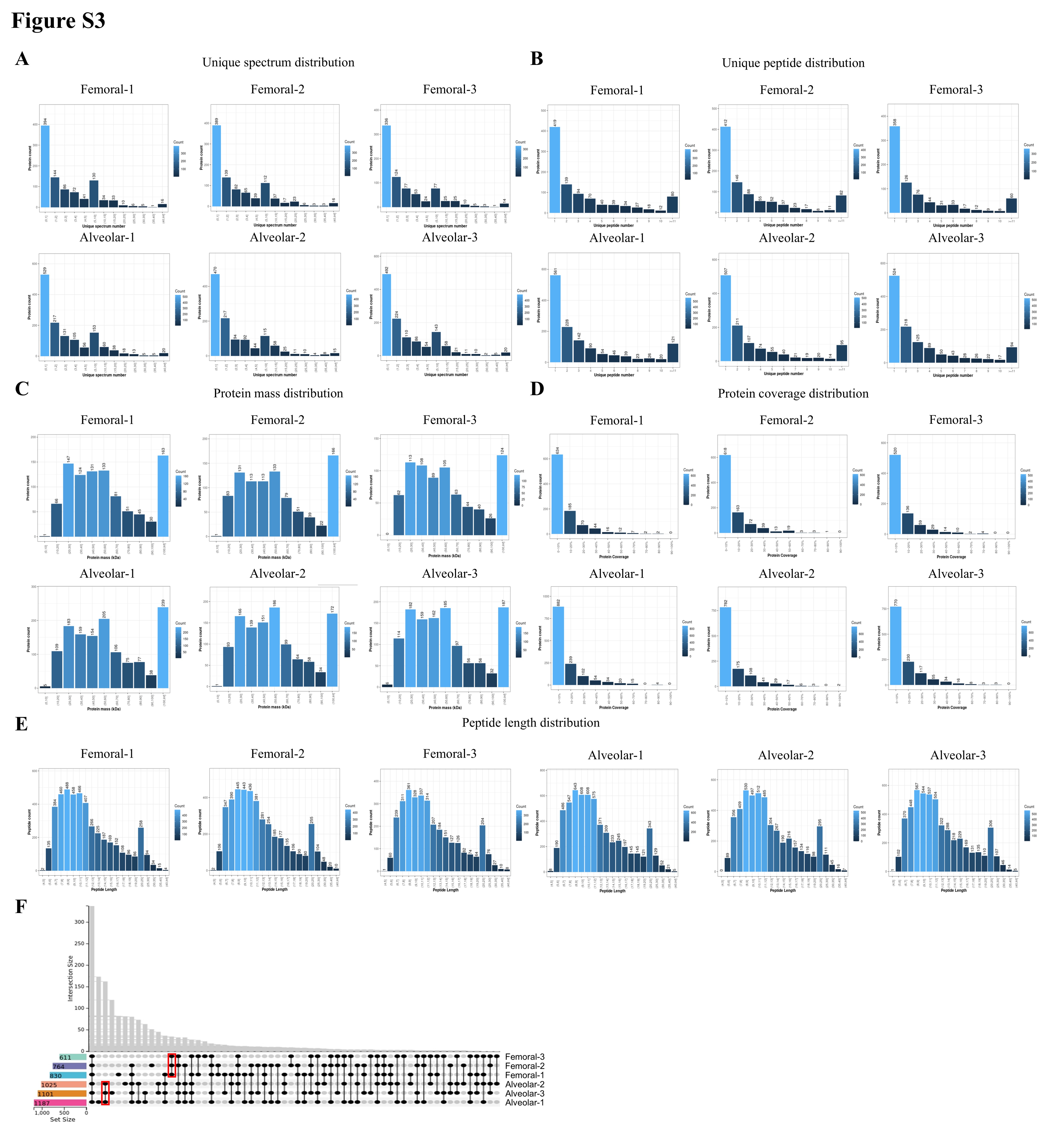


**Fig. S3** Protein identification in early repair tissues of femoral and alveolar bone defects. (**A**-**E**) Unique spectrum distribution, unique peptide distribution, protein mass distribution, protein coverage distribution, and peptide length distribution in early repair tissues of femoral and alveolar bone defects. (**F**) Screening of specific proteins in early repair tissues of femoral (721 proteins) and alveolar (427 proteins) bone defects.


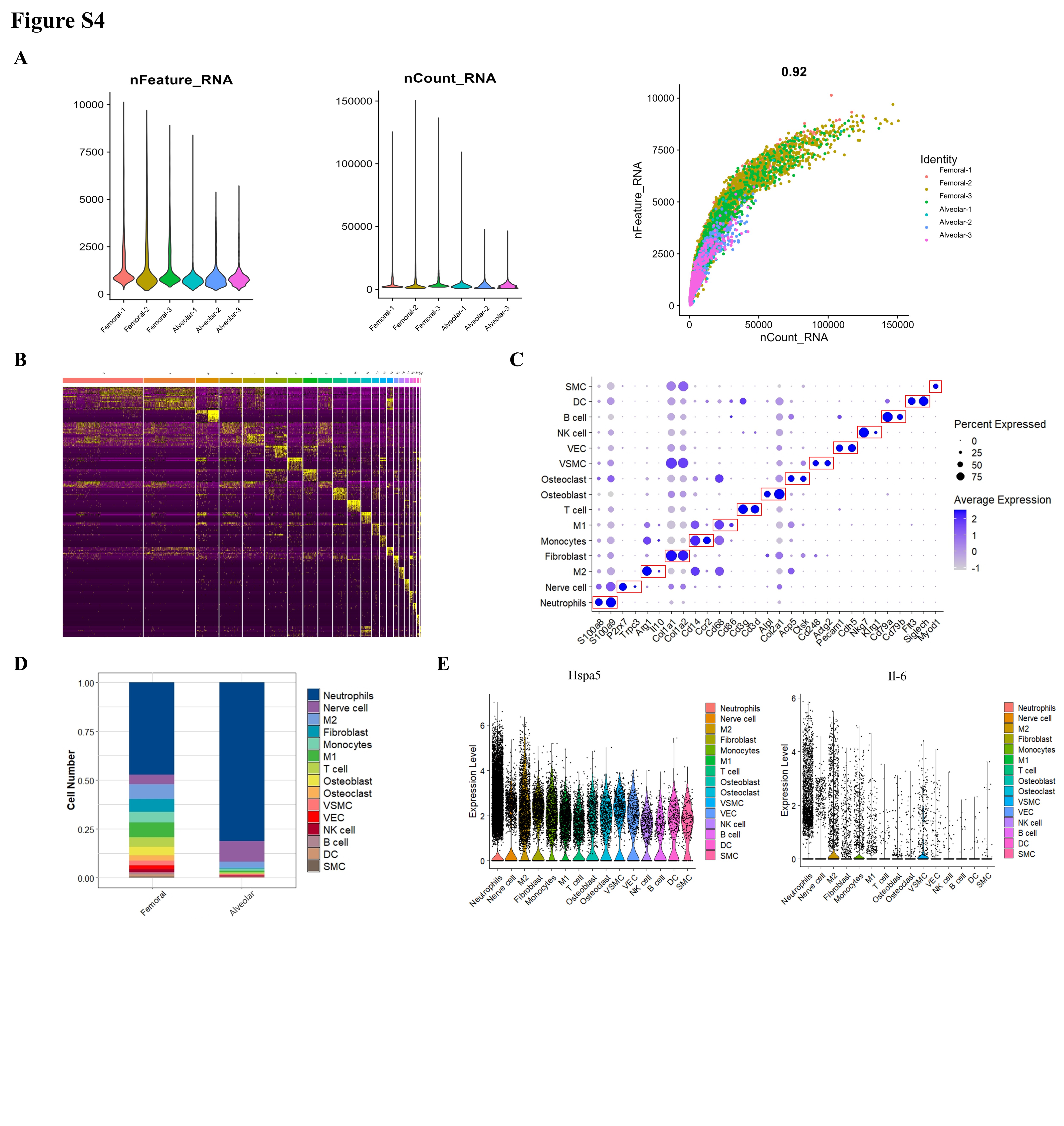


**Fig. S4** Identification of cell populations and gene-cell localization in scRNA-seq data of early tissue in rat bone defects. (**A**) Quality control of scRNA-seq data, including nFeature_RNA, nCount_RNA, and the correlation between these two metrics. (**B**) Heatmap of clustering for the characteristic genes of different cellular clusters. (**C**) Cell population identification. (**D**) Stacked bar chart of cellular proportions of different cell populations in early tissue of femoral and alveolar bone defects. (**E**) Cellular expression localization of *Hspa5* and *Il-6*.


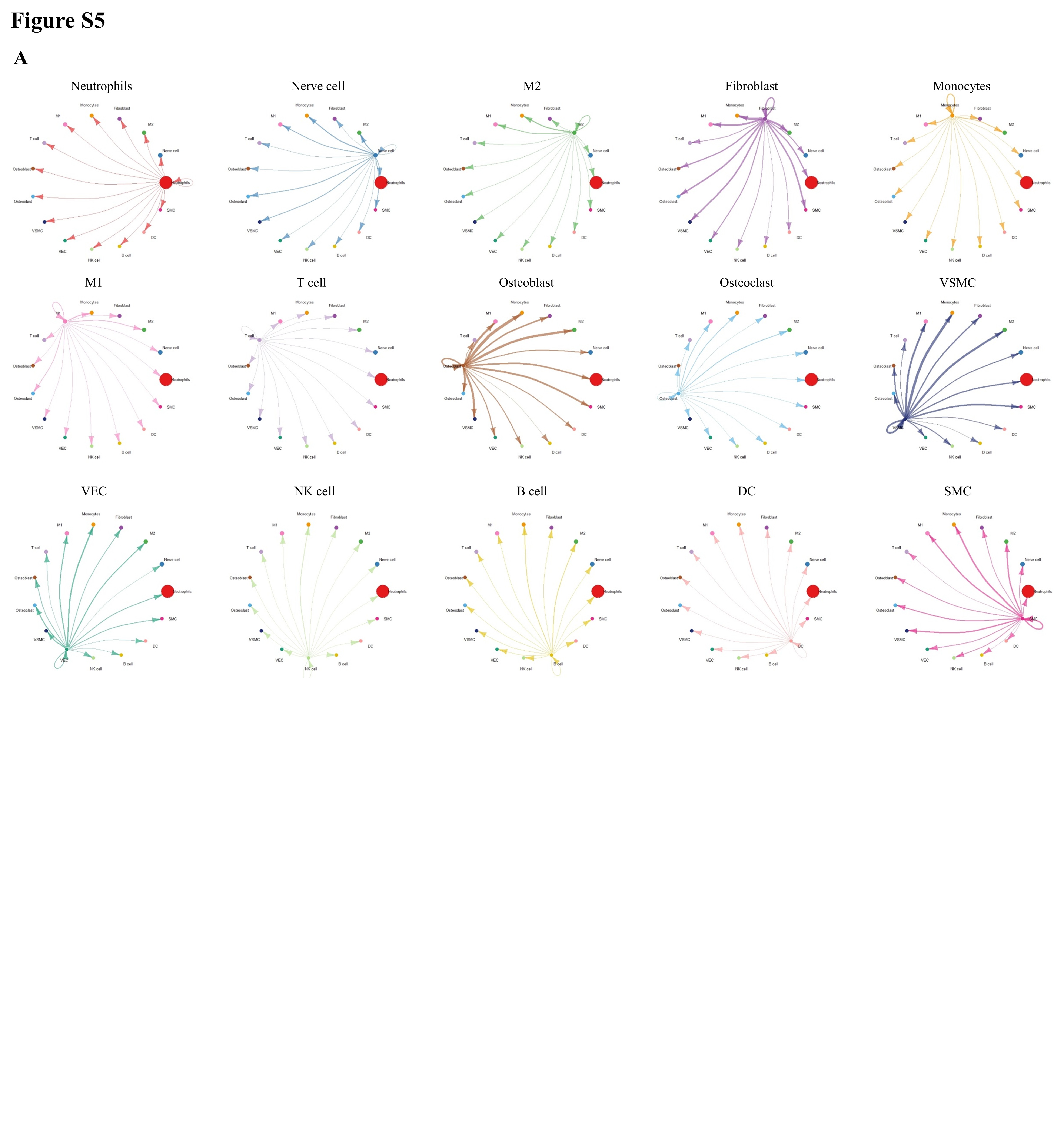


**Fig. S5** Intercellular crosstalk landscape. (**A**) Characterization of intercellular communication among multiple cell types within the early repair tissue of femoral and alveolar bone defects.


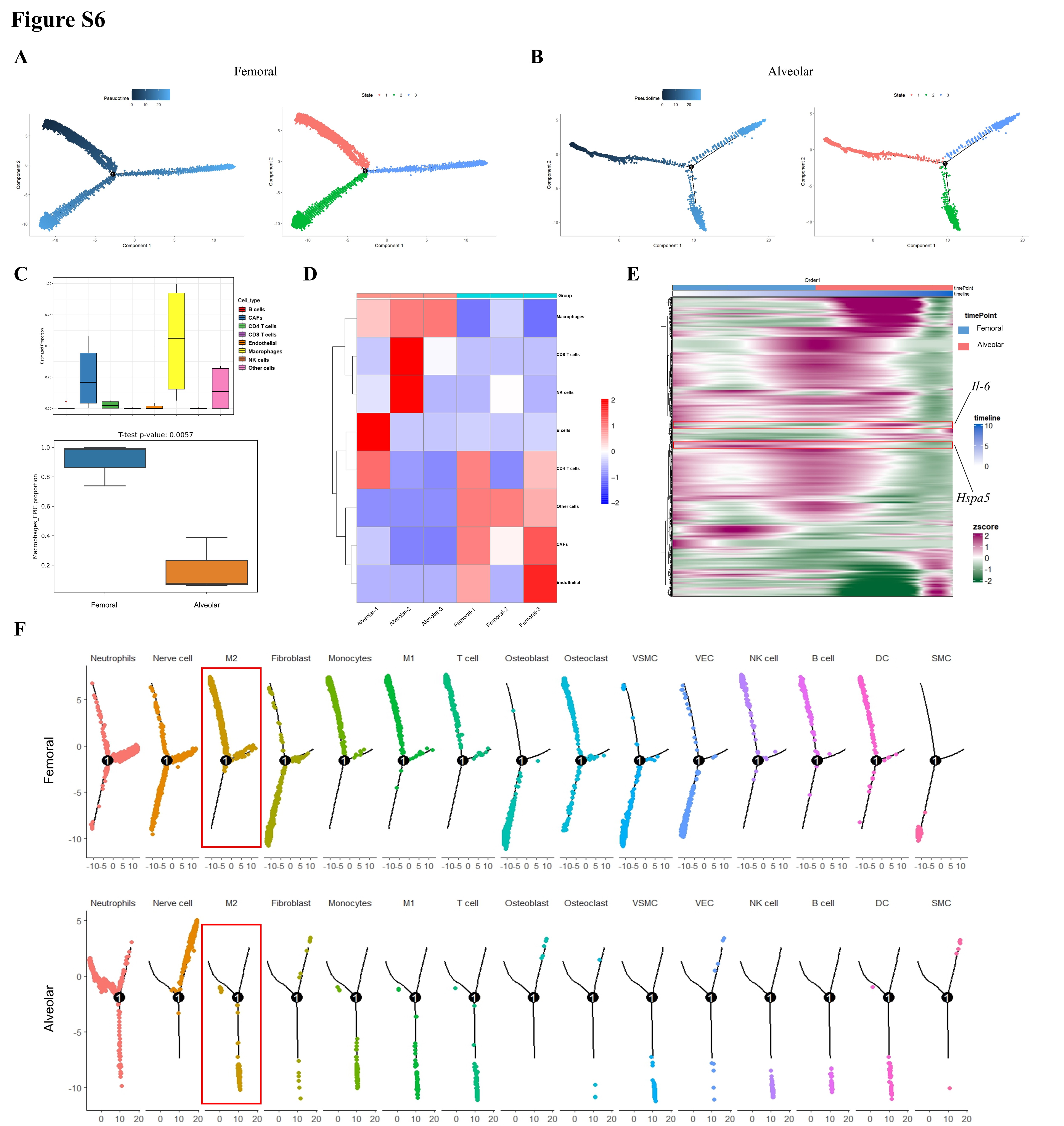


**Fig. S6** Temporal differentiation trajectories of cells and gene expression. (**A**) Cellular differentiation trajectories of early tissue in femoral defects. (**B**) Cellular differentiation trajectories of early tissue in alveolar bone defects. (**C**) Immune infiltration analysis and macrophage levels based on bulk RNA-seq data. (**D**) Grouped immune infiltration heatmap based on bulk RNA-seq data. (**E**) Temporal expression profiles of *Hspa5* and *Il-6* across groups in bulk RNA-seq data. (**F**) Distribution of distinct cell types across temporal trajectories in early tissues of femoral and alveolar bone defects.


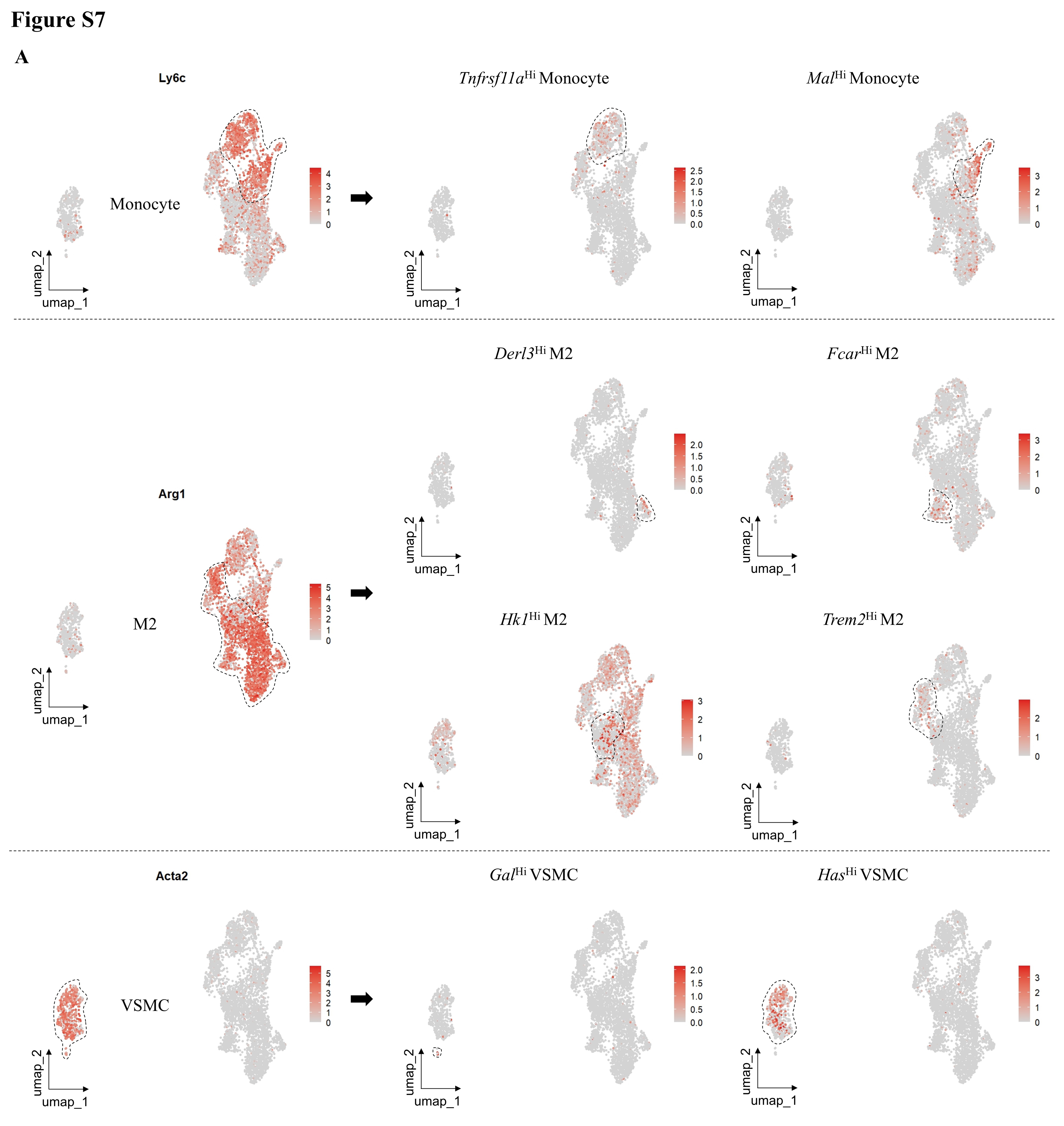


**Fig. S7** Cell Subpopulation-Specific Gene Expression. (**A**) UMAP plot demonstrates that the gene marker is indicative of non-differentiation within cell subpopulations.


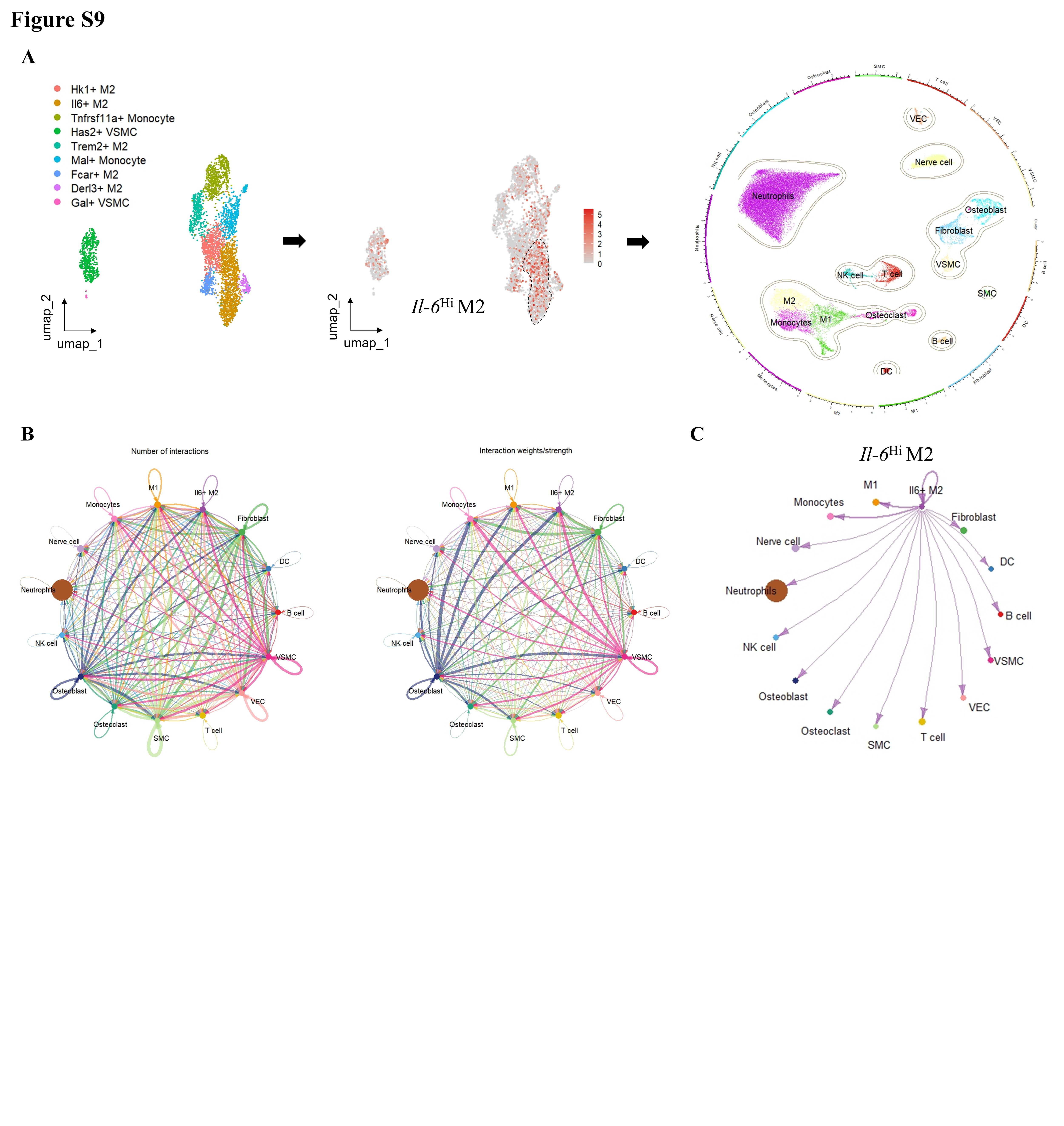


**Fig. S8** Communication between subpopulation cells and other cell groups. (**A**) Isolation of *Il-6*^Hi^ M2 macrophage cell subpopulation and integration with the cell population. (**B**) Number of interactions and interaction weights/strengths among different cells after integration. (**C**) Communication between *Il-6*^Hi^ M2 macrophage cell subpopulation and other cell types.


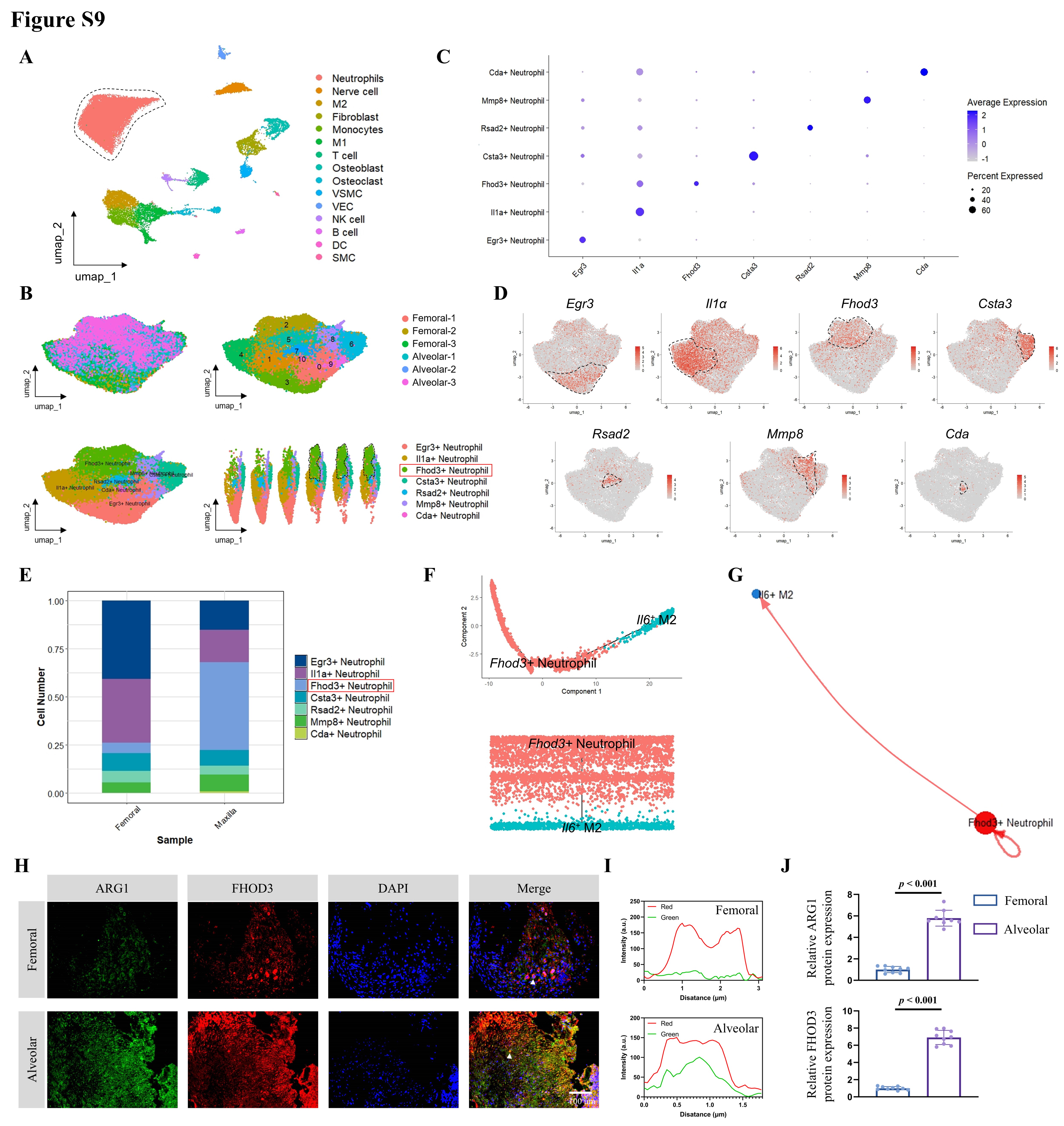


**Fig. S9** *Fhod3*^+^ neutrophils regulate *Il-6*^Hi^ M2 macrophage cells in the early stage of alveolar bone defects. (**A**) Extraction of Neutrophils. (**B**-**D**) Subpopulation and identification of gene-specific neutrophils. (**E**) Stacked bar chart of cellular proportions of neutrophil subsets. (**F**) Temporal trajectory distribution of *Fhod3*^+^ neutrophils and *Il-6*^Hi^ M2 macrophage cells. (**G**) Communication between *Fhod3*^+^ neutrophils and *Il-6*^Hi^ M2 macrophage cells. (**H**-**J**) Immunofluorescence detection of the fluorescent intensity and tissue localization relationship between *Fhod3*^+^ neutrophils and *Il-6*^Hi^ M2 macrophage cells. Arrow, characteristic cell. Scale bars, 100 μm. Statistical significance was determined using t-test.


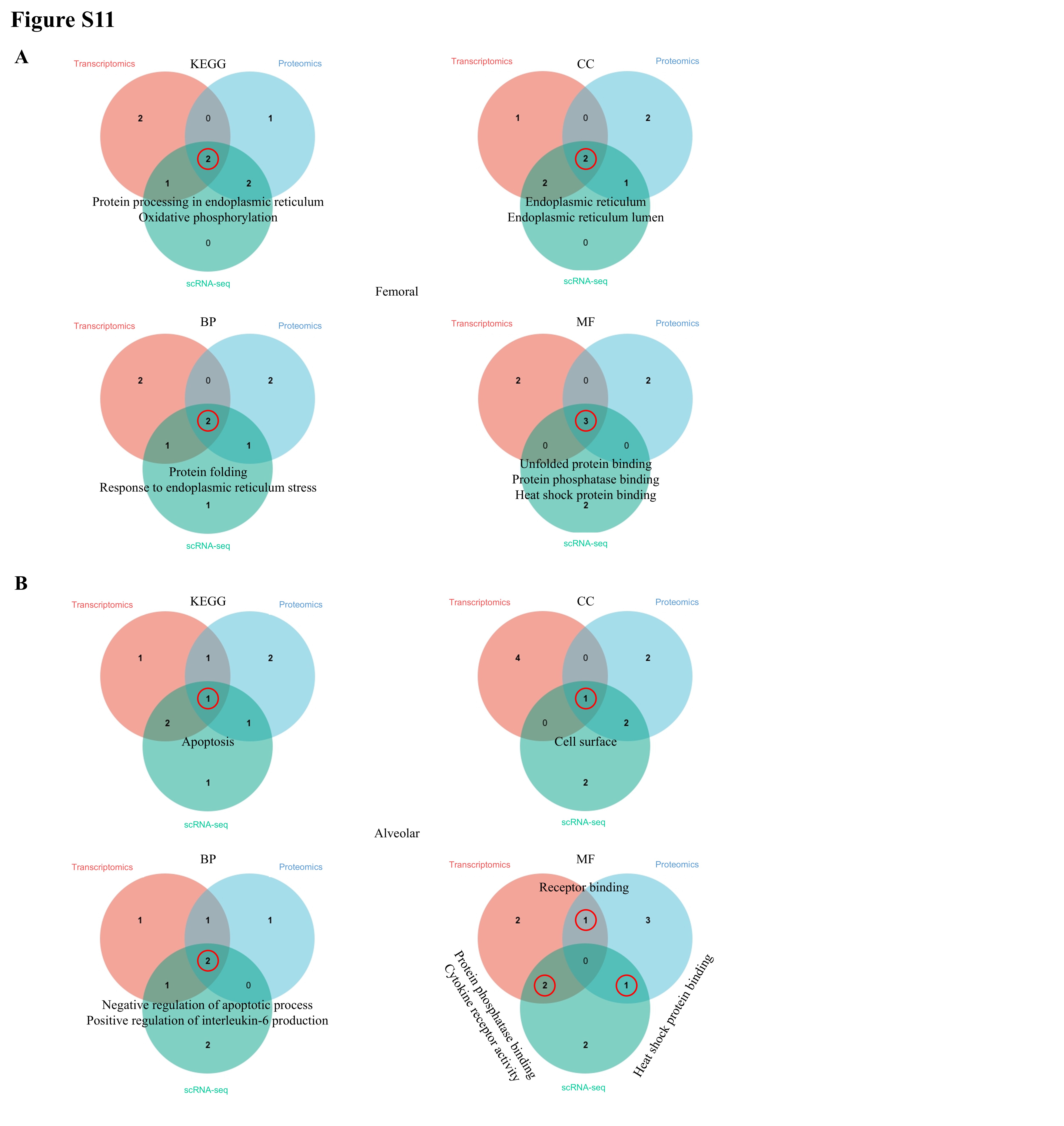


**Fig. S10** Joint analysis of KEGG and GO pathways in early tissue repair of femoral and alveolar bone defects using a multi-omics approach. (A) Integrated analysis of KEGG and GO (BP, biological processes; CC, cellular components; MF, molecular functions) in early-stage femoral bone defects. (B) Integrated analysis of KEGG and GO (BP, biological processes; CC, cellular components; MF, molecular functions) in early-stage alveolar bone defects.


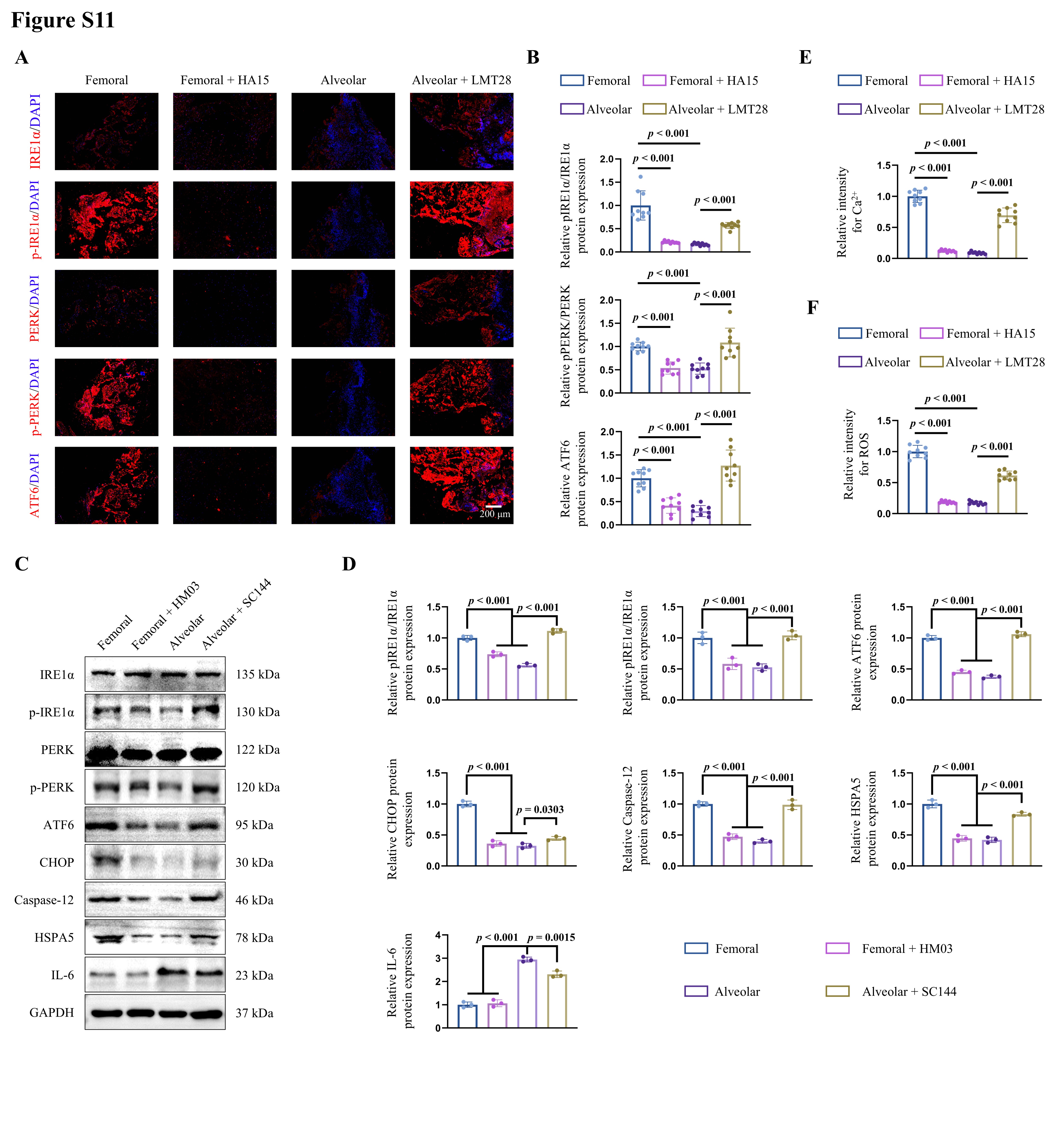


**Fig. S11** IL-6 modulates HSPA5 to inhibit endoplasmic reticulum stress. (**A**, **B**) Immunofluorescence assay was employed to detect the expression levels of endoplasmic reticulum stress-related proteins and conduct quantitative analysis. Scale bars, 200 μm. (**C**, **D**) Expression levels and statistical analysis of endoplasmic reticulum stress (ERS)-related and apoptosis-related proteins in femoral and alveolar bone repair tissues following HM03 and SC144 treatment. (**E**, **F**) Quantitative analysis of Ca^2+^ and reactive oxygen species (ROS) content. Statistical analysis was performed with one-way ANOVA.


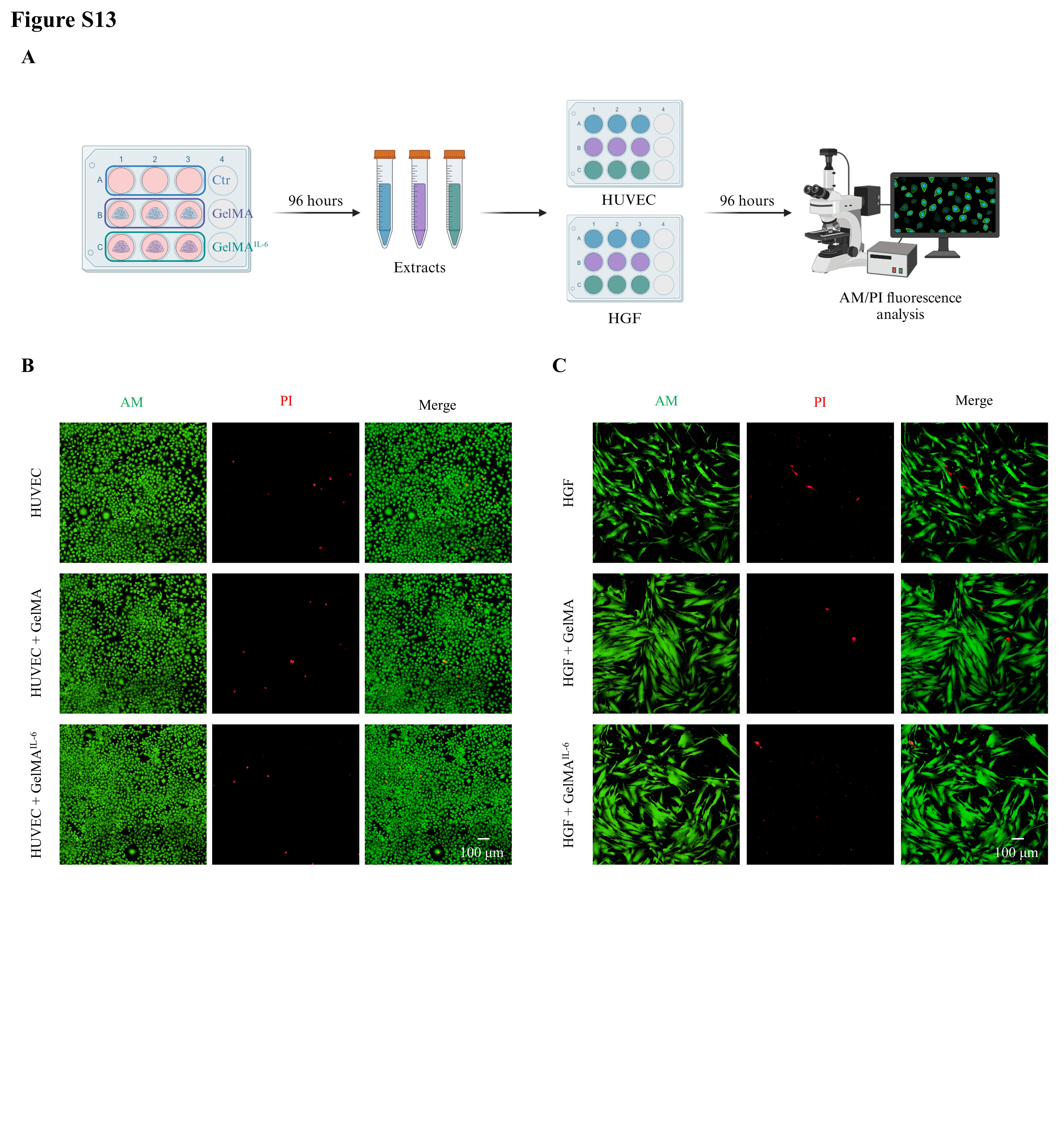


**Fig. S12** Cytotoxicity assessment of GelMA^IL-6^ hydrogel. (**A**) Illustration of cell culture for AM/PI assay. (**B**) Assessment of live and dead status of HUVECs stimulated by GelMA^IL-6^ hydrogel using Calcein-AM/PI assay. Scale bars, 100 μm. (**C**) Assessment of live and dead status of HGF cells stimulated by GelMA^IL-6^ hydrogel using Calcein-AM/PI assay. Scale bars, 100 μm. HUVECs, human umbilical vein endothelial cells. HGF, human gingival fibroblasts. AM, fluorescent dyes for living cells. PI, dead cell fluorescent dye.


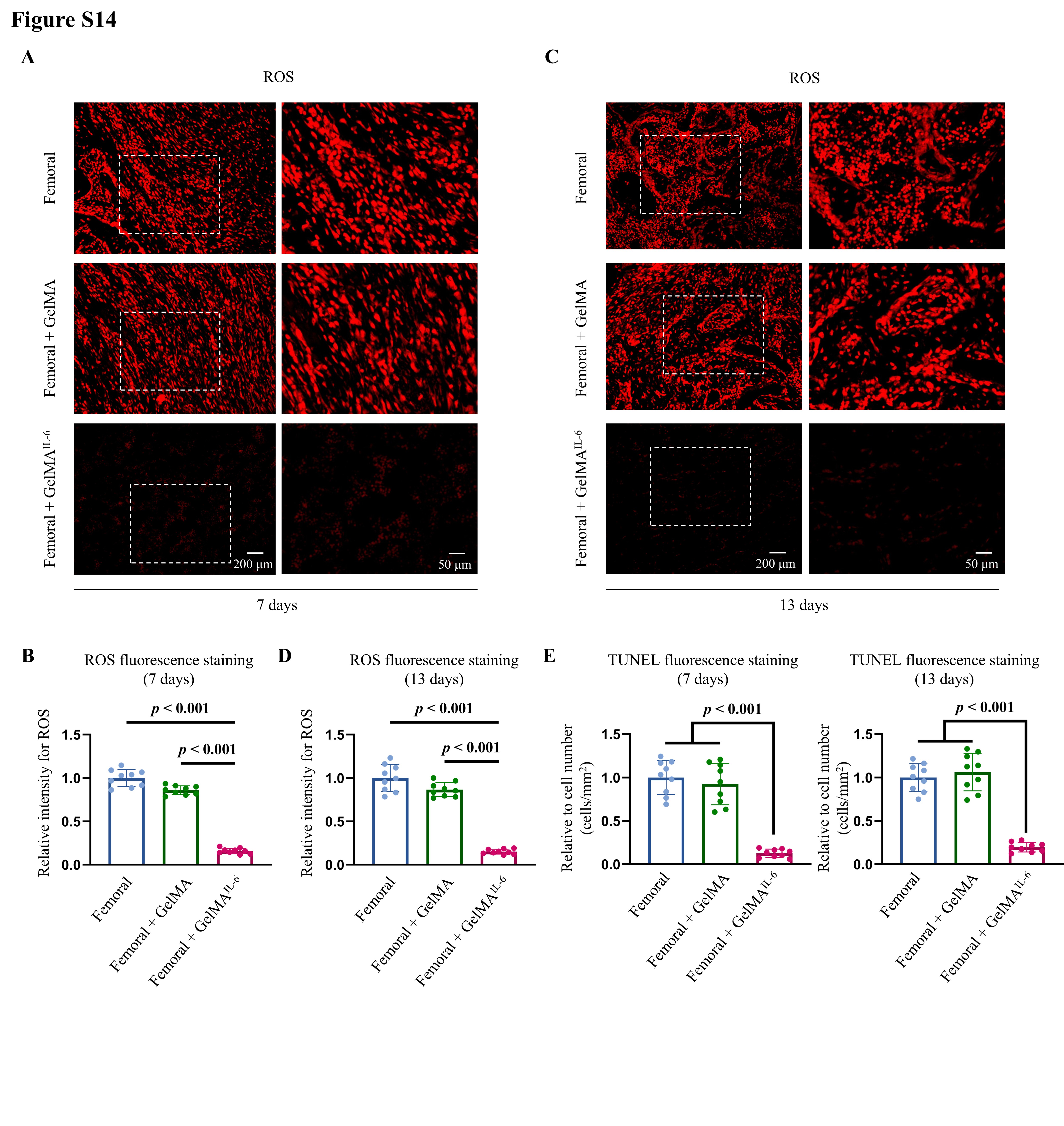


**Fig. S13** Detection of ROS in femoral tissue following GelMA^IL-6^ hydrogel treatment. (**A**, **B**) Quantitative analysis and detection of ROS in tissue at 7 days post-femoral defect. Scale bars, 200 μm and 50 μm. (**C**, **D**) Quantitative analysis and detection of ROS in tissue at 13 days post-femoral defect. Scale bars, 200 μm and 50 μm. (**E**) Statistical analysis of apoptotic cell counts based on terminal deoxynucleotidyl transferase dUTP nick-end labeling (TUNEL) staining. Statistical analysis was performed with one-way ANOVA.

**Supplementary table.** Primer sequences.

| Gene Symbol | Species | Biotype | Primer | Sequence (5′ to 3′) |
| --- | --- | --- | --- | --- |
| *Hspa5* | Rat | mRNA | Forward | CTGTGAGACACCTGACCGAC |
|  |  |  | Reverse | CCGTGCCTACATCCTCCTTC |
| *Cstd* | Rat | mRNA | Forward | CAAGCAGCCTGGAGTCGTAT |
|  |  |  | Reverse | GTGCCGCCAAGCATTAGTTC |
| *Atp6v1h* | Rat | mRNA | Forward | TGTGAACACCTTCGTCGCTAT |
|  |  |  | Reverse | CTTGGCGAGTTTCTCTTTCCG |
| *Il-6* | Rat | mRNA | Forward | CCAGTTGCCTTCTTGGGACT |
|  |  |  | Reverse | CTGGTCTGTTGTGGGTGGTA |
| *Il-10* | Rat | mRNA | Forward | CAGCAAAGGCCATTCCATCC |
|  |  |  | Reverse | TTGGCAACCCAAGTAACCCT |
| *Il-17a* | Rat | mRNA | Forward | CCATGTGCCTGATGCTGTTG |
|  |  |  | Reverse | GTTATTGGCCTCGGCGTTTG |
